# Development, screening, and analysis of DNA aptamer libraries potentially useful for diagnosis and passive immunity of arboviruses

**DOI:** 10.1186/1756-0500-5-633

**Published:** 2012-11-13

**Authors:** John G Bruno, Maria P Carrillo, Alicia M Richarte, Taylor Phillips, Carrie Andrews, John S Lee

**Affiliations:** 1 Operational Technologies Corporation, 4100 NW Loop 410, Suite 230, San Antonio, TX, 78229, USA; 2 U.S. Army Medical Research and Materiel Command, Military Infectious Disease Research Program, 504 Scott Street, Bldg. 722, Fort Detrick, MD, 21702, USA

**Keywords:** Aptamer, Arbovirus, Consensus, DNA, Enzyme-linked, Fluorescence, Lateral flow, SELEX

## Abstract

**Background:**

Nucleic acid aptamers have long demonstrated the capacity to bind viral envelope proteins and to inhibit the progression of pathogenic virus infections. Here we report on initial efforts to develop and screen DNA aptamers against recombinant envelope proteins or synthetic peptides and whole inactivated viruses from several virulent arboviruses including Chikungunya, Crimean-Congo hemorrhagic fever (CCHF), dengue, tickborne encephalitis and West Nile viruses. We also analyzed sequence data and secondary structures for commonalities that might reveal consensus binding sites among the various aptamers. Some of the highest affinity and most specific aptamers in the down-selected libraries were demonstrated to have diagnostic utility in lateral flow chromatographic assays and in a fluorescent aptamer-magnetic bead sandwich assay. Some of the reported aptamers may also be able to bind viral envelope proteins in vivo and therefore may have antiviral potential in passive immunity or prophylactic applications.

**Results:**

Several arbovirus DNA aptamer sequences emerged multiple times in the various down selected aptamer libraries thereby suggesting some consensus sequences for binding arbovirus envelope proteins. Screening of aptamers by enzyme-linked aptamer sorbent assay (ELASA) was useful for ranking relative aptamer affinities against their cognate viral targets. Additional study of the aptamer sequences and secondary structures of top-ranked anti-arboviral aptamers suggest potential virus binding motifs exist within some of the key aptamers and are highlighted in the supplemental figures for this article. One sequence segment (ACGGGTCCGGACA) emerged 60 times in the anti-CCHF aptamer library, but nowhere else in the anti-arbovirus library and only a few other times in a larger library of aptamers known to bind bacteria and rickettsia or other targets. Diagnostic utility of some of the aptamers for arbovirus detection in lateral flow chromatographic assays and a fluorescent sandwich assay on the surface of magnetic microbeads is also demonstrated.

**Conclusions:**

This article catalogues numerous DNA aptamer sequences which can bind various important pathogenic arboviruses and have, in some cases, already demonstrated diagnostic potential. These aptamer sequences are proprietary, patent-pending, and partially characterized. Therefore, they are offered to the scientific community for potential research use in diagnostic assays, biosensor applications or for possible passive immunity and prophylaxis against pathogenic viruses.

## Background

The self-professed aim of BMC Research Notes is to “reduce the loss suffered by the research community when results remain unpublished because they do not form a sufficiently complete story to justify the publication of a full research article.” Such is the case with the following body of aptamer sequence, screening, and structural data for aptamers developed against recombinant proteins or synthetic peptides and whole inactivated viruses that were developed as part of several Small Business Innovative Research (SBIR) contracts with the U.S. Defense Department (see the Acknowledgments section for contract numbers) for diagnostic applications. We have assembled a large body of arboviral aptamer sequence data that has demonstrated initial diagnostic utility, but also has the potential to serve numerous other diagnostic and therapeutic applications beyond that which our small group can currently manage.

The aptamer DNA sequences have been divulged in a U.S. patent application (No. 13/199,082, Publication No. 2012/0149889 A1). While some of the sequences demonstrated diagnostic potential in our enzyme-linked aptamer sorbent assay (ELASA [1,2]), we hypothesize that the highest affinity and most specific aptamers in the screened libraries may also have therapeutic, prophylactic, or further diagnostic potential since aptamers have demonstrated antiviral potential in humans and animals against influenza [3-5] and a number of other viruses [6,7] beginning with HIV in the earliest days of aptamer research [8]. Aptamers may represent a valuable “bridge to life” if used for passive immunity much like intravenous antisera for rapidly acting venoms that kill hosts before an immune response can be induced by a vaccine. Passive aptamer therapy would also hold the advantage of low to nonexistent immunogenicity, because nucleic acids are generally nonimmunogenic and do not lead to allergic reactions or serum sickness upon subsequent administration [9,10].

Arboviruses represent a loosely defined group of mostly RNA viruses from several different families including Bunyaviridae, Flaviviridae, and Togaviridae that share the characteristic of being transmitted to humans or other hosts by arthropods. Around the world, some arboviruses transmitted by mosquitoes, ticks and other arthropods represent a significant threat to human health by induction of potentially epidemic encephalitis or hemorrhagic fevers and other possibly lethal syndromes (e.g., dengue shock syndrome). It is estimated that there are up to 100 million cases of dengue fever worldwide each year and of these cases 250,000 are cases of dengue hemorrhagic fever with 10% mortality [11]. Similarly, Chikungunya virus (CHIKV) infected one-third of the inhabitants of islands in the Indian ocean and at least 1 million people on mainland India between 2005–2006 [11-13]. Crimean-Congo Hemorrhagic Fever (CCHF) virus is a lesser known, but highly lethal Nairovirus and a member of the Bunyaviridae family which is transmitted by *Hyalomma* and other genera of ticks and has a broad geographic distribution across the Eurasian and African continents [14-16]. Tickborne encephalitis virus (TBEV), subtypes European or Western tick-borne encephalitis virus, Siberian tick-borne encephalitis virus, and Far-Eastern tick-borne encephalitis virus (formerly known as Russian spring summer encephalitis virus), are additional tick-vectored viruses with serious health implications [17,18]. Finally, West Nile virus (WNV) has begun posing a significant threat to the U.S. with hundreds to thousands of cases emerging in America every year since 1999 [19].

With the exceptions of Yellow Fever virus, Japanese encephalitis virus and TBEV, no effective or licensed vaccines exist for most arboviruses [18,20-23]. Even the effective vaccines have drawbacks and can require long vaccination times before seroconversion in the recipient, during which time the recipient may be susceptible to the viral infection. Therefore, we considered developing aptamers for both rapid diagnostics and potential therapy or prophylaxis for arbovirus infections. As aforementioned, anti-envelope or anti-nucleocapsid DNA and RNA aptamers have demonstrated efficacy in blocking or inhibiting influenza, hepatitis and other viruses *in vitro*[3-7]. There is also a rich literature concerning aptamer development against HIV surface proteins and reverse transcriptase over the last two decades [8]. These facts, coupled with the commercial availability of many arbovirus envelope or nucleocapsid recombinant proteins and synthetic peptides derived from amino acid sequence data contained within public protein databases (e.g., entries in Table 1), made pursuit of aptamers against these otherwise exotic and dangerous viral targets quite feasible. Ultimately, our aptamer candidates will need to be tested in biosafety level 3 (BSL-3) or BSL-4 level laboratories, but our initial aptamer development using recombinant envelope proteins and synthetic peptide or inactivated whole virus targets was very productive.

**Table 1:** Viral protein and peptide targets used for anti-arbovirus DNA aptamer development

**Virus protein or peptide**	**Molec. Wt.**	**Commercial sources and amino acid sequence of peptides and applicable references**
Chikungunya E1a Peptide	5,605 daltons (amino acids 200–250)	GenScript, Inc. (Piscataway, NJ): GDIQSRTPES KDVYANTQLVLQRPAVGTVHVPYSQAPSGF KYWLKERGAS; Santhosh et al. [13] and Pubmed Protein Sequence Database
CCHF Altamura Gn 611	1,641 daltons	GenScript; **TQ**EGRGHV**KL**SRGSE Altamura et al., 2007 [16]
CCHF 11E7a	3,391 daltons	GenScript; GLKFASLTCTGCYACSSGISCKVRIHVDEPDE Ahmed, et al., 2005 and Altamura et al. 2007 [15,16]
CCHF 11E7b	4,482 daltons	GenScript; VAASSSLMARKLEFGTDSTFKAFSAMPKTSLCFYIVEREY Ahmed, et al., 2005 and Altamura et al. 2007 [15,16]
CCHF 11E7c	3,152 daltons	Genscript; ED**TQ**KCVNT**KL**EQPQSILIEHKGTIIGK Ahmed, et al., 2005 and Altamura et al. 2007 [15,16]
Recombinant dengue Type 1	41 kD E (envelope) antigen	Virostat, Inc. (Portland, ME) Product No. 8812
Recombinant dengue Type 2	15 kD E antigen	Virostat; Product No. 8813
Recombinant dengue Type 3	15 kD E antigen	Virostat; Product No. 8814
Recombinant dengue Type 4	15 kD E antigen	Virostat; Product No. 8815
TBEV CE/gE		Feldan Bio Corp. (Quebec, Canada) Cat. No. FB03-80-149
WNV E Protein	~ 42 kD E protein	GenWay Biotech (San Diego, CA) Cat. No. 10-511-248224

## Materials and methods

### **Synthetic peptide, recombinant protein, and whole virus targets and magnetic bead**

#### Immobilization

Table 1 presents data on the commercial sources for recombinant viral envelope proteins or synthetic peptide amino acid sequences for putative epitopes on envelope proteins used in aptamer development with corresponding references, if available. Recombinant protein or peptide targets (20 μg of each) were immobilized using 100 μl of stock Dynal M280 (2.8 μm diameter; Invitrogen Corp., Carlsbad, CA) tosyl-coated magnetic beads (MBs) for 2 hr at 37°C with gentle mixing according to the manufacturer’s instructions. MBs were then collected using a Dynal MPC-S magnetic rack and washed in 1 ml of 1X binding buffer (1XBB; 0.5 M NaCl, 10 mM Tris–HCl, and 1 mM MgCl_2_, pH 7.5-7.6) three times before storage at 4°C.

In addition to the targets listed in Table 1, some CCHF aptamer development utilized formalin-fixed whole virus particles consisting of the IbAr 10200 and Drosdov (Dros) strains of CCHF (U.S. Army Medical Research Institute for Infectious Disease; USAMRIID; Ft. Detrick, MD). All CCHF virus samples were certified as inactive by USAMRIID after negative plaque assay results using mammalian (CER or Vero) cells and an apparent lack of infectivity based on immunofluorescence microscopy. Inactivated virus (100 μl) was covalently attached to 100 μl of stock tosyl-M280 Dynal MBs for 2 hr at 37°C, followed by washing and storage at 4°C in 1XBB. Protein, peptide, or whole virus-coated MBs (hereafter referred to as target-MBs) were used for DNA aptamer development according to the MB-SELEX procedures previously published by Bruno et al. [1,2] and briefly summarized below.

### Aptamer development, cloning and sequencing

All DNA oligonucleotides were purchased from Integrated DNA Technologies, Inc. (Coralville, IA). MB-based SELEX (Systematic Evolution of Ligands by Exponential enrichment) was performed using 150 nanomoles of the degenerate SELEX template library sequence: 5^′^-ATCCGTCACACCTGCTCT-N_36_-TGGTGTTGGCTCCCGTAT-3^′^, where N_36_ represents the randomized 36-base region of the DNA library. Primer sequences were: 5^′^-ATACGGGAGCCAACACCA-3^′^ (designated forward or F) and 5^′^-ATCCGTCACACCTGCTCT-3^′^ (designated reverse or R) to prime the template and nascent strands, respectively. The random library was reconstituted in 500 μl of sterile nuclease-free water and heated to 95°C for 5 min to ensure that the DNA library was completely single-stranded (ss) and linear. The hot template solution was added to 100 μl of target-MBs (~ 2 X 10^7^ beads) and 600 μl of sterile 2X Binding Buffer (2XBB) and mixed at room temperature (RT; ~ 25°C) for 1 hr.

Following interaction with the randomized DNA libraries, DNA-target-MB complexes were separated from unbound DNA by magnetic collection and the supernate was discarded. DNA-target-MBs were then washed three times in 400 μl of sterile 1XBB with magnetic collection. DNA-target-MBs (~ 75 μl) were added to separate PCR reactions to amplify the bound DNA as follows. The MB pellet was split into 15 μl aliquots and added to five Easy Start™ Micro 50 PCR tubes (Molecular BioProducts, Inc., San Diego, CA), which contained most of the nonperishable components of a PCR reaction beneath a wax seal. A total of 3 μl of 1:10 primer mix (10% forward primer plus 10% reverse primer) in nuclease-free deionized water or ~ 20 nanomoles of each primer per ml plus 1 μl (5 U) of Taq DNA polymerase (Fisher Scientific Inc., Pittsburgh, PA) were added to each of the five PCR tubes which were brought to a final volume of 50 μl each with nuclease-free deionized water. The final MgCl_2_ concentration was 2 mM. PCR tubes were supplemented with 0.5 μl of Perfect Match™ *E. coli* single-strand binding protein (SSBP, Stratagene Inc., La Jolla, CA) to inhibit high molecular weight concatamer formation. PCR was carried out as follows: an initial 95°C phase for 5 min, followed by 30 cycles of 1 min at 95°C, 1 min at 53°C, and 1 min at 72°C followed by a 72°C completion stage for 7 min, and refrigeration at 4°C. This constituted the first of 5 to 10 rounds of MB-SELEX.

CCHF envelope peptides were subjected to 10 rounds of MB-SELEX while all other targets were subjected to 5 rounds. To begin the second round of MB-SELEX and all subsequent rounds, 4 complete tubes of the 5 original PCR tubes were heated to 95°C for 5 min to release bound DNA target-MBs. The fifth tube was always retained and refrigerated as a back-up for that round of the SELEX process. DNA supernate (25 μl per tube) was siphoned out of the hot tubes without removing the target-MBs before the tubes cooled significantly and the DNA was pooled. One hundred μl of hot DNA was added to 100 μl of fresh target-MBs in 200 μl of 2XBB and allowed to mix for 1 hr at RT. Thereafter, the selection and amplification process was repeated for four more rounds of target-MB SELEX with visual verification of 72 bp aptamer PCR products by ethidium bromide-stained 2% agarose electrophoresis after each round. Round 5 or 10 aptamers were cloned into chemically competent and thawed *E. coli* using a GC cloning kit (Lucigen Corp., Middleton, WI) according to the manufacturer’s instructions. Positive clones were sent to Sequetech Corp. (Mountain View, CA) for proprietary rolling circle amplification (RCA)-based sequencing with betaine, DMSO and high heat treatment using an ABI 3730 XL automated sequencer. A comprehensive list of all DNA aptamer sequences related to this work is given in Additional file 1: Table S1. In addition, these sequences were deposited in the U.S. Patent and Trademark Office’s database (http://www.uspto.gov) as part of U.S. patent application No. 13/199,082.

### Enzyme-linked aptamer sorbent assay (ELASA) affinity screening

One hundred μl of 1:300 diluted stock CCHF IbAr 10200 and Drosdov strain formalin-fixed viruses or 250 ng of target proteins or peptides were adsorbed in DNAase-free 96-well flat bottom polystyrene microtiter plates (Greiner Bio-One, GmbH, Frickenhausen, Germany, Product No. 655101) in 100 μl of 0.1M NaHCO_3_ buffer (pH ~ 8.5) per well, covered and stored overnight at 4°C. For screening 250 ng of envelope protein or peptide was immobilized per well. Wells were decanted and washed 3 times in 250 μl of 1XBB for 5 min per wash with gentle mixing and decanted. Wells were then blocked with 2% ethanolamine in 0.1M NaHCO_3_ at 37°C for 1 hr and washed three more times in 1XBB as before. Plates containing lyophilized 5’-biotinylated aptamers (4.5 nanomoles per well) from Integrated DNA Technologies were rehydrated for 1 hr in 100 μl of 1XBB per well with gentle mixing and transferred to the microtiter plate according to a pre-defined map. Aptamers were allowed to interact with targets on the surface of microtiter wells for 1 hr at RT with gentle mixing. Wells were then washed three times in 250 μl of 1XBB for 5 min per wash and decanted. One hundred μl of 1:2,000 streptavidin-peroxidase at 1 mg/ml stock from Thermo Scientific, Inc. (Pittsburgh, PA; Product No. 21126) in 1XBB was added per well with gentle mixing at RT for 30 min. Wells were decanted and washed three more times in 200 μl of 1XBB with gentle mixing for 5 min per wash. Wells were developed by adding 100 μl of One-Step ABTS (Kirkegaard Perry Labs, Gaithersburg, MD) which had been pre-warmed to RT) and read after a 15 min development time or until an O.D. (absorbance) at 405 nm of 1.0 to 2.0 was reached at using a Thermo Scientific microplate reader. Averages of two or four replicate ELASA runs per target are listed in Tables 2, 3 and 4.

**Table 2:** ELASA affinity rankings for anti-chikungunya (ChE), tick-borne encephalitis virus (TBEV), and west nile virus (WNV) aptamers

**Aptamer**	**Avg. A405**	**Aptamer**	**Avg. A405**	**Aptamer**	**Avg. A405**
ChE-17R	2.608	TBEV-2R	2.719	WNV-19F	2.607
ChE-20R	2.583	TBEV-2F	2.440	WNV-18R	2.372
ChE-19R	2.582	TBEV-8R	2.428	WNV-16F	2.363
ChE-16F	2.549	TBEV-6R	2.264	WNV-10F	2.349
ChE-19F	2.462	TBEV-6F	2.238	WNV-12R	2.276
ChE-18F	2.428	TBEV-4R	2.210	WNV-20F	2.186
ChE-17F	2.418	TBEV-1R	2.193	WNV-20R	2.043
ChE-20F	2.412	TBEV-5F	2.123	WNV-16R	1.970
ChE-16R	2.411	TBEV-7F	1.929	WNV-3/7/11F	1.816
ChE-18R	2.343	TBEV-7R	1.922	WNV-3/7/11R	1.764

**Table 3:** ELASA affinity rankings for anti-dengue (DE) serotype 1–4 aptamers

**Aptamer**	**Avg. A405**	**Aptamer**	**Avg. A405**	**Aptamer**	**Avg. A405**	**Aptamer**	**Avg. A405**
DE1-8R	2.651	DE2 - 2R	1.985	DE3 - 1R	1.156	DE4 - 7R	1.910
DE1 - 3R	2.552	DE2 - 10F	1.759	DE3 - 3Ra	0.782	DE4 - 4R	1.497
DE1-10F	2.455	DE2 - 8R	1.671	DE3 - 4Fa	0.760	DE4 - 4F	1.281
DE1-8F	2.404	DE2 - 5Fa	1.641	DE3 - 3Rb	0.744	DE4 - 9Fa	1.269
DE1 -6R	2.321	DE2 - 7F	1.606	DE3 - 2F	0.732	DE4 - 9Rb	1.241
DE1-10R	2.124	DE2 - 2F	1.599	DE3 - 3Fb	0.730	DE4 - 6R	1.228
DE1 - 4R	2.087	DE2 - 10R	1.572	DE3 - 3Fa	0.716	DE4 - 5R	1.193
DE1 - 5Rb	1.906	DE2 - 7R	1.462	DE3 - 4Rb	0.703	DE4 - 3F	1.178
DE1 - 9R	1.886	DE2 - 6R	1.451	DE3 - 4Ra	0.682	DE4 - 9Fb	1.171
DE1 - 3F	1.748	DE2 - 9R	1.426	DE3 - 1F	0.676	DE4 - 7F	1.157

**Table 4:** ELASA affinity rankings for anti-crimean congo hemorrhagic fever (CCHF) aptamers

**Aptamer**	**Avg.A405**	**Aptamer**	**Avg. A405**	**Aptamer**	**Avg. A405**	**Aptamer**	**Avg. A405**	**Aptamer**	**Avg. A405**
Gn6-25R	1.940	E7a-23F	2.230	E7b-1bR	2.069	E7c-23/25R	2.121	Dros-13R	1.267
Gn6-16cF	1.903	E7a-33R	2.123	E7b-8a/10/16-19/23-25R	2.000	E7c-7bF	2.018	Dros-4-7/10R	1.249
Gn6-18F	1.860	E7a-5R	2.084	E7b-1aR	1.999	E7c-27F	2.001	Dros-17R	1.230
Gn6-17F	1.824	E7a-18F	2.082	E7b-3R	1.936	E7c-7aF	1.998	Dros-17F	1.227
Gn6-30F	1.754	E7a-11R	2.073	E7b-14R	1.899	E7c-1bF	1.994	Dros-13F	1.167
Gn6-5F	1.751	E7a-33F	2.070	E7b-1bF	1.875	E7c-4aR	1.988	Dros-16R	1.159
Gn6-7bR	1.688	E7a-29F	2.056	E7b-6R	1.864	E7c-17F	1.982	Dros-16F	1.140
Gn6-16cR	1.668	E7a-20R	2.037	E7b-5R	1.857	E7c-17R	1.958	Dros-4-7/10F	1.136
Gn6-6R	1.639	E7a-8R	2.036	E7b-1aF	1.826	E7c-19F	1.948	Dros-19F	1.103
Gn6-15R	1.633	E7a-20F	2.017	E7b-20/21R	1.781	E7c-24R	1.932	Dros-19R	0.920

### Aptamer-based lateral flow (LF) chromatographic assay development

Aptamers have been successfully used in LF formats by other researchers [24,25]. We fashioned LF strips from a combination of a Whatman GB002 sample pad, a Whatman Standard 17 conjugate pad, a Millipore High Flow 240 analytical membrane (for slower 240 second migration and greatest sensitivity) and a Whatman 470 wicking or absorbent pad all attached on a pressure sensitive sticky laminate backing (Diagnostic Consulting Network; DCN, Carlsbad, CA). The sample pad was soaked in 0.05M Tris–HCl (pH 8.03) containing 0.15 mM NaCl and 0.25% Triton X-100 for 30 min followed by drying of the sample pad strip at 37°C for several hrs until completely dry. The components were assembled with the sample pad overlapping the conjugate pad by ~ 2 mm and both the conjugate pad and wicking pads were overlaid on the nitrocellulose analytical membrane at each end of the analytical membrane. Thereafter, 4 mm wide strips were cut with a sharp paper cutter and these were laid into plastic cassettes (DCN) for evaluations.

One hundred μl of 10 O.D. units/ml of colloidal gold-streptavidin conjugate from DCN was bound to 100 μl of 5’-biotin-aptamers (ranging from 1.33 to 1.83 mg/ml with an average molec. wt. of ~ 22.5 kD) which had been preheated to 95°C for 5 min followed by rapid cooling at -20°C for 5 min. Each aptamer-biotin-streptavidin-colloidal gold conjugate was added to 250 μl of 1XBB buffer and mixed in sterile microfuge tubes for 1 hr at RT. Fifty μl of 1 μM biotin was added to block any unbound streptavidin binding sites and all aptamers were purified through 30 kD MWCO (molecular weight cut off) spin columns from PALL Life Sciences (Ann Arbor, MI) by spinning at 5,000 r.p.m. on an Eppendorf Mini-Spin Plus microfuge for 30 min. The colloidal gold-aptamer conjugate was resuspended in 100 μl of 1XBB off of the spin column membrane. Capture aptamer-streptavidin reagents for the test lines (or dots) were made in precisely the same manner as the reagents for the conjugate pads except that the streptavidin was not labeled with colloidal gold. One μl of the capture aptamer-streptavidin reagent was spotted onto the analytical membrane at various distances from the conjugate pad, allowed to air dry and then baked in a UV oven for 15 min (total imparted energy ~ 0.2 J/cm^2^). Fifteen μl of each conjugate aptamer-biotin-streptavidin-colloidal gold reagent were added to the conjugate pads and air dried for lateral flow experiments using ~ 1 μg of recombinant envelope protein or synthetic peptide.

### Spectrofluorometric assay and fluorescence microscopic verification of a CCHF aptamer magnetic bead sandwich assay

**Figure 1:**
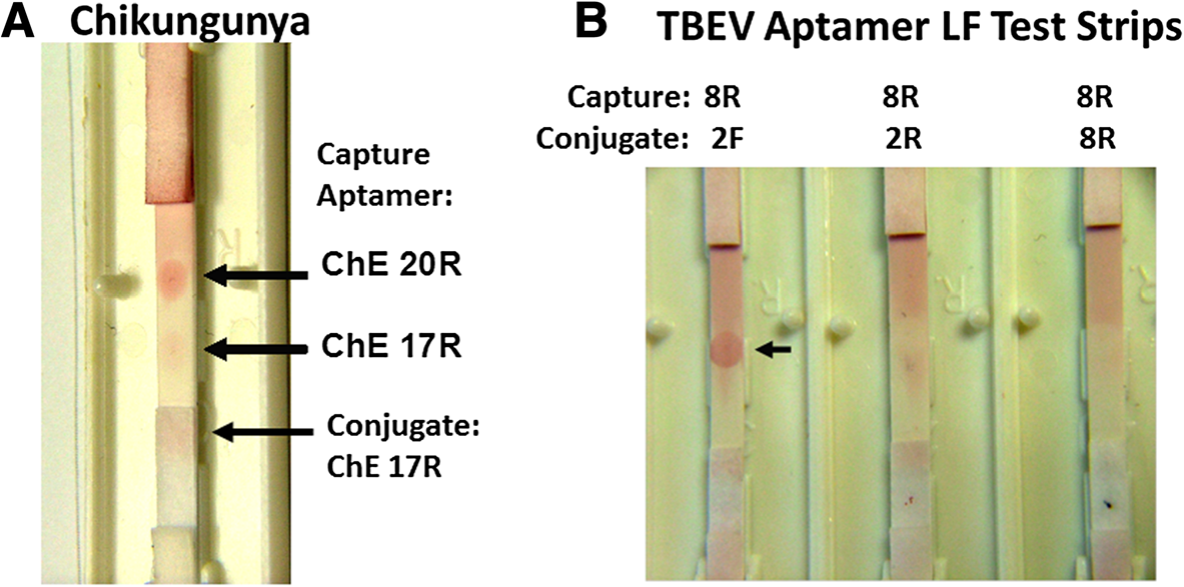
**Preliminary aptamer LF chromatographic assay experiments for (A) Chikungunya virus envelope peptide detection and (B) Tick-borne encephalitis virus (TBEV) recombinant envelope protein detection.** Capture and conjugate aptamers are shown for each test strip and ~ 1 μg each target peptide or protein in PBS was used for these tests which were allowed to develop for 5 min at RT. High Flow (HF) 240 analytical membranes were used in each case.

μl of capture aptamer-5’-biotin-streptavidin-magnetic beads (~ 1.3 X 10^6^ M270 Dynal/Life Technologies, Inc. MBs per test) that had been washed 3 times in 1 ml of 1XBB on a Dynal MPC-S magnetic collection rack. The capture aptamer-MB reagents were combined with 20 μl (~ 2 μg) of 5’-TYE 665 dye-5’-reporter aptamers (Integrated DNA Technologies, Inc., Ex/Em 645/658-660 nm) per test. Preliminary testing (not shown) indicated that these were the optimal reagent amounts to maximize assay sensitivity. For screening purposes (Figure 1), we used 150 ng of the inactivated CCHF virus per test (as determined by a Bradford protein assay) which was performed in triplicate. Titration studies using two-fold serial dilutions of the inactivated CCHF virus starting with 150 ng of virus per test were conducted in PBS using the most highly fluorescent combination 20 (Gn6-25R capture aptamer-MBs plus Gn6-17F-TYE 665 reporter aptamer) sandwich assay. Fluorescence spectra and emission peak heights at 658 nm were obtained by collecting all of the captured virus on aptamer-MBs with a small cylindrical magnet placed inside a Cary Varian Eclipse spectrofluorometer to hold the MBs in place with 2 ml of PBS in polystyrene cuvettes. The spectrofluorometer was set to excite at 645 nm with 5 nm slits and emission spectra were scanned from 655 to 720 nm using a photomultiplier tube (PMT) setting of 900 V.

Fluorescence microscopy was performed to verify virus binding at a total magnification of 400X. Digital images were taken with an Olympus BH-2 fluorescence microscope equipped with a Semrock (TDEX Corp., Lake Forest, IL) filter cube customized for use with TYE 665 fluorophore and a Moticam 2000 (2.0 Mega pixel) digital camera (Motic Instruments, Inc., Richmond, British Columbia, Canada) and Motic image capture software. Images were analyzed objectively using NIH Image Java (Image J) software to generate histograms of red fluorescence intensity from the surface of MBs to determine if fluorescence intensity from the TYE 665 dye was increased in the presence of 150 ng of inactivated CCHF virus.

#### Secondary structure determinations

Two dimensional stem-loop structures were generated for comparison of common loops or potential binding pockets or sites using free internet-based Vienna RNA software as described by Hofacker [26], except that DNA parameters and a temperature setting of 25°C were used. Vienna RNA software is publicly accessible at: http://rna.tbi.univie.ac.at/cgi-bin/RNAfold.cgi. All secondary structures are shown in the supplemental figures. While common loop structures do not necessarily indicate a target binding site within a family of aptamers, they do represent clearly accessible single-stranded regions and are probably more energetically favorable for target binding versus “induced fit” in double-stranded stem regions wherein the aptamer would have to be opened by breaking hydrogen bonds between the nitrogen bases.

## Results and discussion

Tables 2, 3, 4 rank the top ten aptamers from each virus group based on ELASA absorbance (average absorbance from two or four separate ELASA experiments) from highest to lowest affinity (greatest to lowest absorbance at 405 nm). It is important to emphasize that some virus groups had many more candidate aptamer sequences as revealed in the comprehensive list (Additional file 1: Table S1). The four different nucleotides are color-coded to facilitate visually identifying identical or similar sequences amidst the complexity in Additional file 1: Table S1. Only the top ten ELASA rankings for each viral target are given in Tables 2, 3, 4. Background absorbance at 405 nm was always < 0.4. The constant sequence18-base flanking primer regions (ATACGGGAGCCAACACCA, AGAGCAGGTGTGACGGAT, ATCCGTCACACCTGCTCT, and TGGTGTTGGCTCCCGTAT) on the 5^′^ and 3^′^ ends were included in Additional file 1: Table S1, because primer regions may contribute to aptamer binding loops or pockets [27] and may possibly stabilize secondary or tertiary aptamer structures. However, our investigations primarily focused on the 36-base region in the middle of each aptamer as it represented the most probable location for aptamer binding due to its randomized nature.

In addition to primary nucleotide sequence comparisons, aptamer DNA sequences were also analyzed for common segments or “runs” present in the predicted single-stranded loop structures based on secondary structures derived from internet-based Vienna RNA software [26] using 25°C and physicochemical parameters associated with DNA instead of RNA. Additional file 2: Figures S1–13 present a great diversity of two-dimensional aptamer shapes that sometimes converged on common structural motifs in whole or in part. This observation underscored the diversity of the starting random library that resulted in the convergence of structure and sequence diversity through the affinity selection and enrichment process known as SELEX. Common sequences residing in or proximal to natural loop structures may represent binding sites and are indicated by the boxed, circled, or underlined sequences in Additional file 2: Figures S1–13. While it is true that induced fit of the target into a double-stranded (ds) stem region of an aptamer is feasible when the change in free energy makes binding favorable, extant single-stranded (ss) loop structures having common sequences are more likely to fold into binding sites in most cases and should be considered first for detailed structural analyses.

Primary nucleotide sequence analysis of the top ten (Table 2) anti-Chikungunya envelope (ChE) aptamers presented in Additional file 1: Table S1 revealed a shared sequence of AACCCGCA in aptamer sequences ChE-17F and ChE-18R. Most of this segment resides in a loop structure in ChE-18R as seen in Additional file 2: Figure S1, but most of this same sequence lies in a ds stem region in ChE-17F. Therefore, if this sequence is part of a binding site, it may require induced fit or other tertiary conformational changes to properly bind ChE. Other than that observation, only one other similar structure was noted between ChE aptamers 18F and 19F in Additional file 2: Figure S1 where it is denoted by boxes around the structures. These structures, albeit odd, involve both 18-base primer regions and are therefore less likely to be involved in aptamer binding [27].

It is interesting to note from Additional file 1: Table S1, that one of the top ten ELASA ranked (Table 2) anti-WNV aptamer sequences emerges three times (WNV 3F/R was identical to clones 7F/R and 11F/R). To appear in three of 17 clones (34 total F and R aptamers) or about 17.6% of the population that started with a diversity of ~ 10^15^ sequences is significant and may represent a consensus sequence that has the ability to bind a WNV envelope protein epitope. In addition, two other sequences in the top ten anti-WNV aptamer list (WNV-10F and -19F) differed by only one base (T versus C) as shown in Additional file 1: Table S1 and Additional file 2: Figure S13. In addition, WNV-10F and -19F were oddly truncated to 59 bases as compared to the normal 72 base length. This may be due to the *E. coli* host cell excising a segment from the inserted aptamer in a plasmid or skipping over a GC-rich “knot” during replication or sequencing.

The anti-dengue envelope (DE) aptamer analysis (Table 3 and Additional file 1: Table S1) was relatively disappointing by comparison to the CCHF analysis. With the exception of a few odd structures contained mostly in the constant primer ends and a few GC-rich loop structures which are circled or boxed in Additional file 2: Figures S8–11, there was no noteworthy convergence to a full-length consensus sequence, sequence segment, or stem-loop motif. The anti-dengue aptamers were spread over four different envelope protein serotypes and only 8 or 9 forward and reverse aptamers were sequenced (16–18 total aptamer sequences per DE serotype for a total of 68 aptamers) as shown in the comprehensive list in Additional file 1: Table S1. Therefore, more aptamer sequencing may be required to define anti-dengue aptamer consensus sequences for each serotype.

Similar to the BE aptamer analysis, relatively few anti-TBEV aptamer sequences were identified (18 total sequences in Additional file 1: Table S1). And similarly, among the top ten anti-TBEV sequences identified by the ELASA screening (Table 2), there was very little convergence to common sequences, segments, or structural motifs as shown in Additional file 2: Figure S12.

No clear consensus or common sequence runs (Additional file 1: Table S1) emerged from analysis of the top anti-CCHF Altamura Gn611 [16] aptamers listed in Table 4, but it is noteworthy that Gn6-25R which ranked first in the ELASA is identical to another top-ranked anti-CCHF sequence (E7c-23/25R) at the top of Tables 4 and 5 as well. Oddly, the Altamura Gn611 peptide and 11E7c peptide (derived from a monoclonal antibody binding site on CCHF) do not share much amino acid sequence identity [15,16]. It appears that the longest homologous segments are two runs of two amino acids (TQ and KL, bolded and underlined in Table 1).

**Table 5:** Identical anti-CCHF aptamer sequences (without flanking primer sequences)

**Aptamer**	**Sequence**
E7c - 23/25R	ACAGTTAGAGCTTGCCGTATGCCTTTGTTAACATAA
Gn6 - 25R	ACAGTTAGAGCTTGCCGTATGCCTTTGTTAACATAA
E7a - 2/6/28/30bR	ACTAACCGAATGGCAGTTTCCCCCTTATCCATCTAT
E7c - 1a/18/20R	ACTAACCGAATGGCAGTTTCCCCCTTATCCATCTAT
Gn6 - 11R	ACTAACCGAATGGCAGTTTCCCCCTTATCCATCTAT
E7a – 15R	GGGATAGGGTCTCGTGCTAGATG
E7b - 13/15R	GGGATAGGGTCTCGTGCTAGATG
E7a - 8R	CGCTGAAGCAAGACATTATCGGGACATTGCCGTGA
E7b - 20/21R	CGCTGAAGCAAGACATTATCGGGACATTGCCGTGA
IbAr 10200 – 2-6/8-11/13-23/25R	TGACACGCGT**ACGGGTCCGGACATGTC**ATAACGGAC
E7a – 3/10/16/19/21/22R	TGACACGCGT**ACGGGTCCGGACATGTC**ATAACGGAC
E7b – 8a/10/16-19/23-25R	TGACACGCGT**ACGGGTCCGGACATGTC**ATAACGGAC

Note: The complementary Forward (F) sequences also match, but were not included for brevity.

Outside of three similar loop structures proximal to the 5^′^ ends of the anti-CCHF 11E7a-5R, -8R and -11R aptamers (boxed in Additional file 2: Figure S3), analysis of the 11E7a aptamer sequences shown in Additional file 1: Table S1 and Additional file 2: Figure S3 did not demonstrate much congruence. However, analysis of the sequences in Additional file 1: Table S1, Additional file 2: Figures S4 and 7, uncovered a common ACGGGTCCGGACA sequence (underlined and bolded in Table 5). This is the longest contiguous sequence common among all of the anti-CCHF aptamers and if the identical clones are counted, this sequence occurs 60 times in the anti-CCHF aptamer library. In many cases, the sequence can be extended to include TGTC on its 3’ end as well. Because this sequence run was so prevalent, we scanned our library of greater than 3,000 aptamer sequences developed against various bacterial, viral and clinical protein or small molecule aptamers and found that this segment is shared with a few anti-*Rickettsia* anti-*Listeria* flagellin and anti-*Salmonella* aptamers as well as one aptamer that binds myoglobin, although the latter may be purely coincidental. The prevalence of this sequence segment and its emergence in rickettsia and bacterial binding populations suggests that it may bind a common epitope involved in attachment or invasion which may have been evolutionarily conserved across some viruses and bacterial cells. The ACGGGTCCGGACA or ACGGGTCCGGACATGTC sequences can be seen associated with loop and stem structures in Additional file 2: Figures S4, 6, and 7 or specifically with top-ranked anti-CCHF aptamer sequences 11E7b-1bR, 11E7b-8aR, Drosdov 4/7/10R (or simply Dros 4R), Dros 13R, and CCHF IbAr 10200 aptamer clones 2-6/8-23/25/26/28/30/31/33/34R. Other identical sequences from the CCHF aptamer population are delineated in Table 5. Unfortunately, due to computational limitations, our analysis is limited to one and two-dimensional aptamer structures and ignores the role of the many possible three-dimensional aptamer-target interactions. However, the reader should consider such 3-D geometries and docking possibilities in any important future analyses, if possible, as our group has done for a few particular cases in the past [28,29].

To demonstrate utility of at least some of the top affinity ranked arbovirus candidate aptamers, we attempted to use the aptamers in a lateral flow chromatographic test strip format [24,25] and a fluorescent sandwich assay format on the surfaces of MBs as has been accomplished with antibody-coated MBs for detection of reoviruses [30]. Figure 1A demonstrates that the Chikungunya envelope aptamers designated 17R (conjugate) and 20R (capture) could be used in a colloidal gold LF sandwich strip format. Interestingly, ChE 17R could not be paired with itself for both the colloidal gold conjugate and capture roles, because it gave only a faint red spot (Figure 1A), suggesting that ChE 17R binds a different epitope on the Ch E1a peptide (Table 1) than does the ChE 20R aptamer (i.e., all of the ChE 17R eptiopes are bound by 17R aptamer and not available for capture by the 17R aptamer). Figure 1B illustrates that TBEV 2F attached to the colloidal gold conjugate and paired with TBEV 8R in the capture dot was a good combination for detection of the TBEV recombinant envelope protein (Table 1). However, when TBEV 8R is paired with itself and competes for the same epitope, detection is not possible (Figure 1B). Similarly, when TBEV 2R is used in the conjugate pad and coupled with TBEV 8R for capture, detection of the recombinant protein failed (Figure 1), perhaps because the TBEV 2R aptamer interferes with capture by 8R on the membrane. Still, the two successful detection dots in Figure 1 demonstrate the feasibility of using some aptamers in an LF strip format for detection of arbovirus envelope proteins.

**Figure 2:**
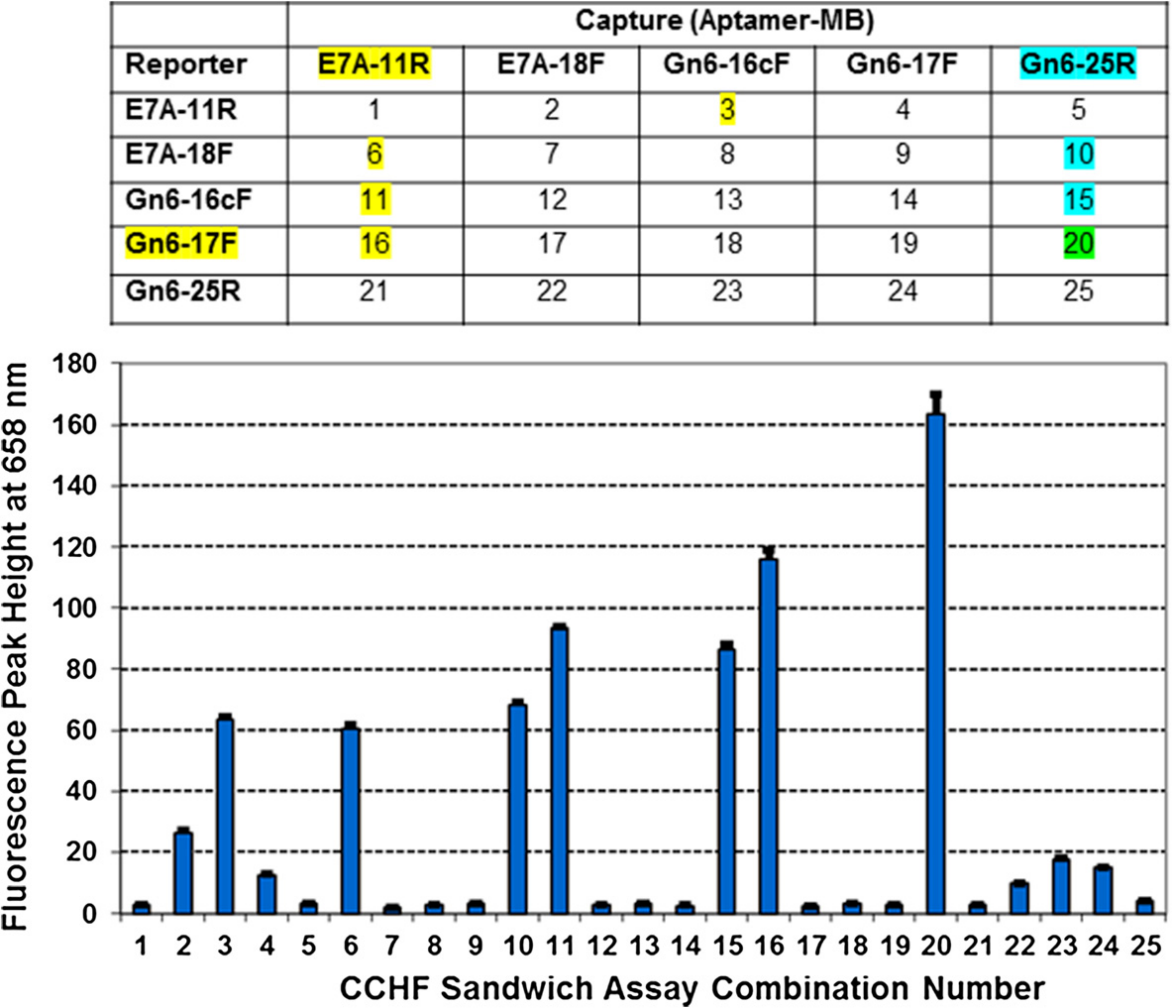
**Top – capture and reporter aptamer sandwich matrix used to screen for the best fluorescent aptamer-MB combination.** Bottom – bar graph showing average peak heights at 658 nm (using TYE 665 fluorophore) for three separate measurements. Error bars represent the range of the three peak heights. Excitation was at 645 nm with 5 nm slits and emissions were scanned from 655 to 720 nm with a photomultiplier (PMT) setting of 900 V.

In another demonstration of diagnostic potential, we used some of our top affinity ranked and best studied CCHF aptamers to devise a preliminary fluorescent aptamer-MB sandwich assay that can be assessed with a spectrofluorometer and verified by use of a fluorescence microscope [30]. Figure 2 shows the results of screening a 5 X 5 capture and reporter sandwich combination matrix against 150 ng of whole formalin-fixed IbAr 10200 CCHF virus using peak height at 658 nm from the fluorescence emission spectra of each sandwich combination. Clearly superior fluorescence detection arose from combination no. 20 using Gn6-25R for capture and Gn6-17F-TYE 665 as the reporter aptamer. It is interesting to note that all of the strongest fluorescent assay combinations in Figure 2 bottom (i.e., combination nos. 10, 11, 15, 16, and 20) involved Gn6-25R or Gn6-17F and E7A-11R (highlighted in the matrix at the top of Figure 2).

**Figure 3:**
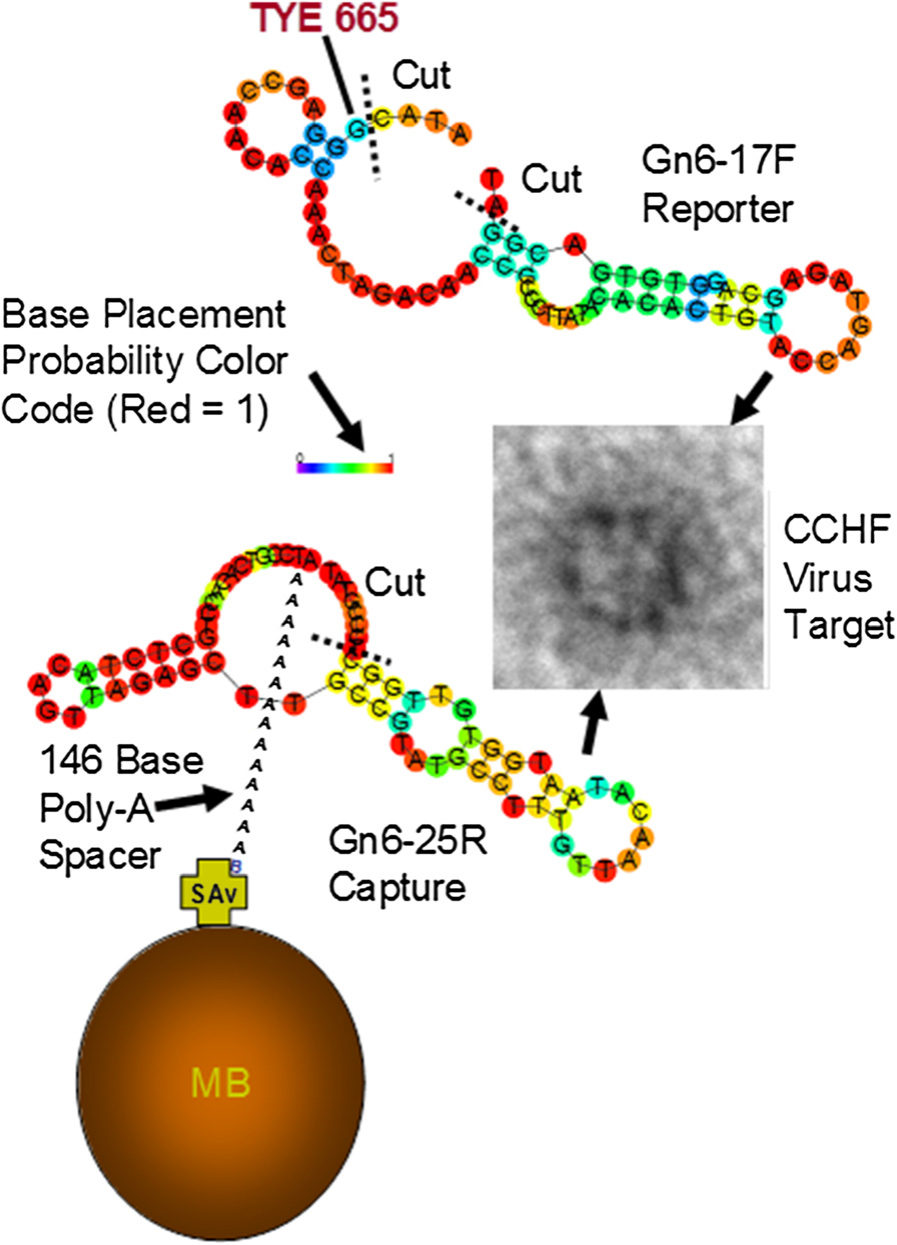
**Schematic of the refined combination 20 CCHF prototype assay showing the 146 poly-adenine spacer tail with a terminal 5’-biotin for coupling to streptavidin (SAv)-coated MBs and the cut sites where the capture and reporter aptamers were truncated during synthesis to prevent partial hybridization in the absence of the virus target, thereby lowering background fluorescence.** The secondary structures of the capture and reporter aptamers were used to determine where the primer regions could hybridize and were truncated.

**Figure 4:**
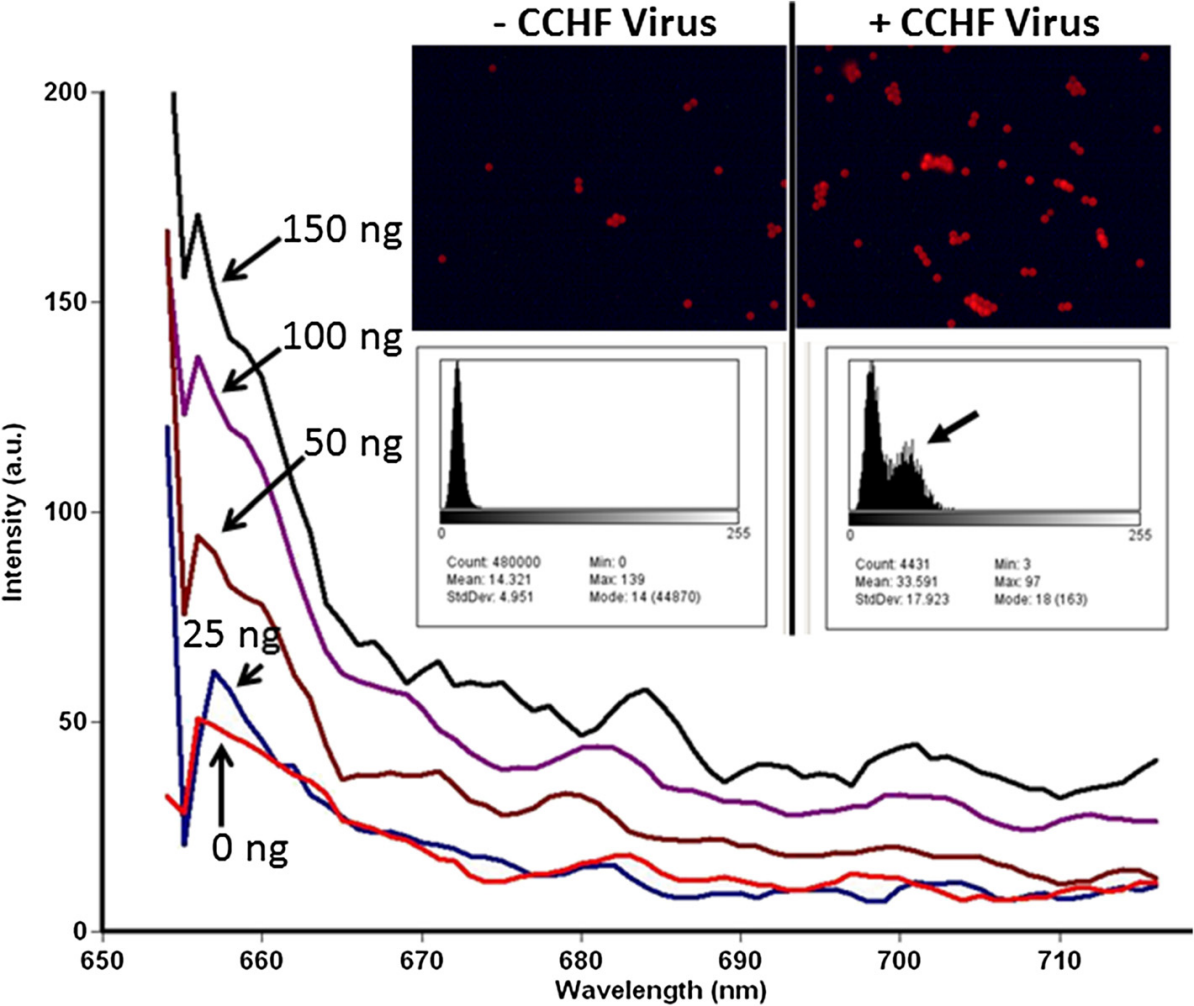
**Results of the two-fold serial dilution experiment using formalin-fixed IbAr 10200 strain of CCHF.** Excitation was at 645 nm with 5 nm slits and emissions were scanned from 655 to 720 nm with a PMT setting of 900 V. The inset shows fluorescence microscopic images captured from the zero control (− CCHF virus) and the 150 ng of inactivated virus (+ CCHF virus) samples scraped from the inside of cuvettes and placed on microscope slides. NIH Image J image analysis software was used to verify that the red TYE 665 fluorescence intensity increased after capture of 150 ng of CCHF virus as illustrated by the histograms in the inset (highlighted by the arrow).

We further refined the CCHF combination 20 assay by truncating a few bases from the ends as shown in Figure 3 to decrease partial hybridization of the capture and reporter aptamers when no target virus is present, thereby lowering background fluorescence. We also lengthened the capture aptamer by adding a 146 poly-adenine tail having a 5’-biotin terminus to connect it to the streptavidin (SAv)-coated MBs (Figure 3). The lengthening of the capture aptamer and truncation of partially hybridizing ends led to marginal improvement in assay sensitivity (data not shown). When this optimized combination 20 assay was used to detect two-fold serial dilutions of formalin-fixed IbAr 10200 CCHF virus using a spectrofluorometer with a magnet in the light path behind the collected assay MBs, it performed well as shown in Figure 4 (inset). Also illustrated in Figure 4 is the fluorescence microscopy of MBs scraped from the inside of the cuvettes from the zero added virus (-CCHF virus) background control versus the + CCHF virus sample which received 150 ng of the inactivated virus and yielded clearly stronger red fluorescence which is visible in the photomicrographs and shows a secondary peak of stronger fluorescence in the NIH Image J-derived histogram (arrow in Figure 4 inset).

## Conclusions

This article presents a potentially valuable catalogue of anti-arbovirus DNA aptamer sequences that may someday aid in diagnostic assays or devices for rapid and sensitive detection of their cognate arbovirus epitopes. In addition, the various families of aptamers may find value for passive immunity or prophylaxis in much the same way that antisera are used to bind and counteract toxins, but without the threat of generating an immune response against the aptamers which are thought to be nonimmunogenic [9,10]. Aptamers should also be less expensive to produce than humanized monoclonal antibodies, especially if their mass production is eventually conducted by PCR or other biological processes instead of parallel chemical synthesis.

Some common full-length or partial sequences were identified for some of the aptamer families and suggest that these sequences are best suited to binding viral envelope proteins. One partial sequence or segment in particular (ACGGGTCCGGACA) emerged in 60 of the anti-CCHF aptamers and bound both viral envelope peptides and whole fixed CCHF viruses, thereby strongly suggesting that this aptamer region may bind to an exposed epitope. That same sequence was often, but not always, associated with an extension of TGTC on its 3’ end. The ACGGGTCCGGACA sequence is not common in the overall library of greater than 3,000 candidate aptamer DNA sequences that our group has amassed over the last ten years, but it can also be found in a few aptamers that bind species of pathogenic bacteria and rickettsia as well as the protein myoglobin. The significance of this finding is ambiguous, but worth reporting for posterity so that future correlations might be made. Other more minor sequence segments of potential interest such as AACCCGCA emerged in two anti-Chikungunya aptamers. Some of these common sequence segments exist in or near secondary loop structures and may represent complete or partial binding sites. In most cases, common sequences or sequence segments ranked in the top ten aptamers following ELASA screening, thereby further solidifying the hypothesis that these sequences could represent binding sites for viral envelope or spike proteins. This hypothesis was further verified when one aptamer was shown to interfere with the binding of a second aptamer in the LF assay prototypes (e.g., Figure 2b, TBEV 2R and 8R aptamers).

Clearly more sequencing, ELASA and other screening methods are needed to clarify some of the questions that have been raised and superficially addressed herein. However, we felt that the already large body of sequence, screening, and structural data warranted release to the scientific community so that other researchers might analyze and build from it as well. Finally, the present work clearly demonstrates diagnostic potential for some of the candidate arbovirus aptamers in LF assay and fluorescent MB sandwich assay formats. We are continuing to use the aptamers reported here in various assay formats for arbovirus detection and hope to use the highest affinity aptamers for *in vitro* (plaque inhibition) and *in vivo* (animal survival) virus challenge studies in the future.

## Abbreviations

1XBB: Single strength aptamer binding buffer; ChE: Chikungunya envelope protein; CCHF: Crimean-Congo Hemorrhagic Fever virus; DE: Dengue envelope protein; Dros: Drosdov strain of CCHF ds, Double-stranded; HF: High Flow membrane; LF: Lateral flow test strip; MBs: Magnetic beads; MWCO: Molecular weight cut off; O.D.: Optical density or absorbance; PMT: Photomultiplier tube; RT: Room temperature (~25°C); SAv: Streptavidin; SELEX: Systematic Evolution of Ligands by Exponential enrichment; ss: Single-stranded; TBEV: Tickborne Encephalitis Virus; WNV: West Nile virus.

## Competing interests

The lead author (JGB) has a financial interest in the sale of some of the aptamers described herein as research reagents available at: http://www.otcbiotech.com.

## Authors’ contributions

JGB, MPC, AMR, TP, and CA carried out experimental procedures. JGB designed and supervised studies. JSL prepared and supplied the formalin inactivated CCHF viruses. JGB and JSL drafted and revised the manuscript. All authors read and approved the final manuscript.

## Supplementary Material

Additional file 1 Comprehensive anti-arbovirus aptamer sequence list. Click here for file

Additional file 2 **Figure S1.** Secondary structures of the top ten ELASA ranked Chikungunya aptamers. **Figure S2.** Secondary structures of the top ten ELASA ranked Crimean-Congo Hemorrhagic Fever (CCHF) Altamura Gn611 aptamers. **Figure S3.** Secondary structures of the top ten ELASA ranked CCHF 11E7a aptamers. **Figure S4.** Secondary structures of the top ten ELASA ranked CCHF 11E7b aptamers. **Figure S5.** Secondary structures of the top ten ELASA ranked CCHF 11E7c aptamers. **Figure S6.** Secondary structures of the top ten ELASA ranked CCHF Drosdov strain whole virus-developed aptamers. **Figure S7.** Secondary structures of consensus CCHF IbAr 10200 strain whole virus-developed aptamers in the entire aptamer library. Note that the reverse sequence contains the most common ACGGGTCCGGACA sequence segment (underlined) in its structure as well. **Figure S8.** Secondary structures of the top ten ELASA ranked dengue serotype 1 aptamers. **Figure S9.** Secondary structures of the top ten ELASA ranked dengue serotype 2 aptamers. **Figure S10.** Secondary structures of the top ten ELASA ranked dengue serotype 3 aptamers. **Figure S11.** Secondary structures of the top ten ELASA ranked dengue serotype 4 aptamers. **Figure S12.** Secondary structures of the top ten ELASA ranked Tick-borne Encephalitis Virus (TBEV) aptamers. **Figure S13.** Secondary structures of the top ten ELASA ranked West Nile Virus (WNV) aptamers. Click here for file

## References

[B1] Bruno JG Kiel JL Use of magnetic beads in selection and detection of biotoxin aptamers by ECL and enzymatic methods Biotechniques 2002 32 178 182 11808691 10.2144/02321dd04

[B2] Bruno JG Carrillo MP Phillips T Edge A Discrimination of recombinant from natural human growth hormone using DNA aptamers J Biomolec Techn 2011 22 27 36 PMC3059541 21455479

[B3] Jeon SH Kayhan B Ben-Yedidia T Arnon R A DNA aptamer prevents influenza infection by blocking the receptor binding region of the viral hemagglutinin J Biol Chem 2004 279 48410 48419 10.1074/jbc.M409059200 15358767

[B4] Cheng C Dong J Yao L Chen A Jia R Huan L Guo J Shu Y Zhang Z Potent inhibition of human influenza H5N1 virus by oligonucleotides derived by SELEX Biochem Biophys Res Comm 2009 366 670 674 18078808 10.1016/j.bbrc.2007.11.183

[B5] Park SY Kim S Yoon H Kim KB Kalme SS Oh S Song CS Kim DE Selection of an antiviral RNA aptamer against hemagglutinin of the subtype H5 avian influenza virus Nucleic Acid Ther 2011 21 395 402 22017542 10.1089/nat.2011.0321

[B6] Kikuchi K Umehara T Nishikawa F Fukuda K Hasegawa T Nishikawa S Increased inhibitory ability of conjugated RNA aptamers against the HCV IRES Biochem Biophys Res Comm 2009 386 118 123 10.1016/j.bbrc.2009.05.135 19501043

[B7] Ellenbecker M Sears L Li P Lanchy JM Stephen Lodmell J Characterization of RNA aptamers directed against the nucleocapsid protein of Rift Valley fever virus Antiviral Res 2012 93 330 339 10.1016/j.antiviral.2012.01.002 22252167 PMC3299919

[B8] Dey AK Griffiths C Lea SM James W Structural characterization of an anti-gp120 RNA aptamer that neutralizes R5 strains of HIV-1 RNA 2005 11 873 884 10.1261/rna.7205405 15923374 PMC1370772

[B9] Healy JM Lewis SD Kurz M Boomer RM Thompson KM Wilson C McCauley TG Pharmacokinetics and biodistribution of novel aptamer compositions Pharm Res 2004 21 2234 2246 10.1007/s11095-004-7676-4 15648255

[B10] Pendergrast PS Marsh HN Grate D Healy JM Stanton M Nucleic acid aptamers for target validation and therapeutic applications J Biomolec Techn 2005 16 224 234 PMC2291729 16461946

[B11] Chahar HS Bharaj P Dar L Guleria R Kabra SK Broor S Co-infections with Chikungunya virus and dengue virus in Delhi, India Emerg Infect Dis 2009 15 1077 1080 10.3201/eid1507.080638 19624923 PMC2744227

[B12] Niedrig M Zeller H Schuffenecker I Drosten C Emmerich P Rumer L Donoso-Mantke O International diagnostic accuracy study for the serological detection of Chikungunya virus infection Clin Microbiol Infect 2009 15 880 884 10.1111/j.1469-0691.2009.02851.x 19624513

[B13] Santhosh SR Dash PK Parida M Khan M Rao PV Appearance of E1:A226V mutant Chikungunya virus in coastal Karnataka, India during 2008 outbreak Virology J 2009 6 172 178 10.1186/1743-422X-6-172 19857273 PMC2774687

[B14] Whitehouse CA Crimean-Congo Hemorrhagic Fever Antiviral Res 2004 64 145 160 15550268 10.1016/j.antiviral.2004.08.001

[B15] Ahmed AA McFalls JM Hoffmann C Filone CM Stewart SM Paragas J Khodjaev S Shermukhamedova D Schmaljohn CS Doms RW Bertolotti-Ciarlet A Presence of broadly reactive and group-specific neutralizing epitopes on newly described isolates of Crimean-Congo Hemorrhagic Fever virus J Gen Virol 2005 86 3327 3336 10.1099/vir.0.81175-0 16298978

[B16] Altamura LA Bertolotti-Ciarlet A Teigler J Paragas J Schmaljohn CS Doms RW Identification of a novel C-terminal cleavage of Crimean-Congo Hemorrhagic Fever virus PreGN that leads to generation of an NSM protein J Virol 2007 81 6632 6642 10.1128/JVI.02730-06 17409136 PMC1900101

[B17] Pletnev AG Yamshchikov VF Blinov VM Tick-borne encephalitis virus genome: the nucleotide sequence coding for virion structural proteins FEBS Lett 1986 200 317 321 10.1016/0014-5793(86)81160-7 3709796

[B18] Craig SC Pittman PR Lewis TE Rossi CA Henchal EA Kuschner RA Martinez C Kohlhase KF Cuthie JC Welch GE Sanchez JL An accelerated schedule for tick-borne encephalitis vaccine: the American military experience in Bosnia Am J Trop Med Hyg 1999 61 874 878 10674662 10.4269/ajtmh.1999.61.874

[B19] Weaver SC Reisen WK Present and future arboviral threats Antiviral Res 2010 85 328 345 10.1016/j.antiviral.2009.10.008 19857523 PMC2815176

[B20] Chang GJ Kuno G Purdy DE Davis BS Recent advancement in flavivirus vaccine development Expet Rev Vaccine 2004 3 199 220 10.1586/14760584.3.2.199 15056045

[B21] Coller BA Clements DE Martyak T Yelmene M Thorne M Parks DE Advances in flavivirus vaccine development IDrugs 2010 13 880 884 21154147

[B22] Ray D Shi PY Recent advances in flavivirus antiviral drug discovery and vaccine development Recent Pat Anti-infect Drug Discov 2006 1 45 55 10.2174/157489106775244055 18221133

[B23] Pulmanausahakul R Khakpoor A Smith DR The development of flavivirus vaccines African J Biotechnol 2010 9 409 415

[B24] Liu J Mazumdar D Lu Y A simple and sensitive “dipstick” test in serum based on lateral flow separation of aptamer-linked nanostructures Ang Chem Int Ed 2006 45 1 5 10.1002/anie.200690000 17094149

[B25] Xu H Mao X Zeng Q Wang S Kawde AN Liu G Aptamer-functionalized gold nanoparticles as probes in a dry-reagent strip biosensor for protein analysis Anal Chem 2009 81 669 675 10.1021/ac8020592 19072289

[B26] Hofacker IL Vienna RNA secondary structure server Nucleic Acids Res 2003 31 3429 3431 10.1093/nar/gkg599 12824340 PMC169005

[B27] Cowperthwaite MC Ellington AD Bioinformatic analysis of the contribution of primer sequences to aptamer structures J Mol Evol 2008 67 95 102 10.1007/s00239-008-9130-4 18594898 PMC2671994

[B28] Bruno JG Carrillo MP Phillips T Hanson D Bohmann JA DNA aptamer beacon assay for C-telopeptide and handheld fluorometer to monitor bone resorption J Fluoresc 2011 21 2021 2033 10.1007/s10895-011-0903-6 21643742

[B29] Bruno JG Carrillo MP Phillips T Vail NK Hanson D Competitive FRET-aptamer-based detection of methylphosphonic acid: a common nerve agent metabolite J Fluoresc 2008 18 867 876 10.1007/s10895-008-0316-3 18224427

[B30] Bruno JG Francis K Ikanovic M Rao P Dwarakanath S Rudzinski W Reovirus detection using immunomagnetic-fluorescent nanoparticle sandwich assays J Bionanosci 2007 1 84 89 10.1166/jbns.2007.015

