# Supplementary material for: Development, screening, and analysis of DNA aptamer libraries potentially useful for diagnosis and passive immunity of arboviruses
Source: BMC Res Notes. 2012 Nov 13;5:633. doi: 10.1186/1756-0500-5-633 (PMC3517355; doi:10.1186/1756-0500-5-633)
Supplement: Additional file 1 — Comprehensive anti-arbovirus aptamer sequence list. [file 1756-0500-5-633-S1.docx]

**Supplemental Table 1 – Comprehensive Anti-Arbovirus Aptamer Sequence List**

**Chikungunya E1a (ChE) Peptide**

ChE -1F

ATACGGGAGCCAACACCACACAGAGGACGGTTCGTCAGATGCCGTTTGCCACGAAGAGCAGGTGTGACGGAT

ChE -1R

ATCCGTCACACCTGCTCTTCGTGGCAAACGGCATCTGACGAACCGTCCTCTGTGTGGTGTTGGCTCCCGTAT

ChE – 2F

ATACGGGAGCCAACACCAAAAAAGGTCTTCTCCCACGATGTGTCCAATGCATCCAGAGCAGGTGTGACGGAT

ChE - 2R

ATCCGTCACACCTGCTCTGGATGCATTGGACACATCGTGGGAGAAGACCTTTTTTGGTGTTGGCTCCCGTAT

ChE – 3F (truncated to 71 bases)

ATACGGGAGCCAACACCACCGCCTCGACCGCTCGGGGCCCTTACCCTAGCTTCAGAGCAGGTGTGACGGAT

ChE – 3R (71 bases)

ATCCGTCACACCTGCTCTGAAGCTAGGGTAAGGGCCCCGAGCGGTCGAGGCGGTGGTGTTGGCTCCCGTAT

ChE – 4F

ATACGGGAGCCAACACCACCACAACCGTAGGGACCCGCCTGGTCCCCAACCTAGAGAGCAGGTGTGACGGAT

ChE – 4R

ATCCGTCACACCTGCTCTCTAGGTTGGGGACCAGGCGGGTCCCTACGGTTGTGGTGGTGTTGGCTCCCGTAT

ChE – 6F

ATACGGGAGCCAACACCACCTCGGATGGTTATGATATAGTTTACAATCATGAAGAGAGCAGGTGTGACGGAT

ChE – 6R

ATCCGTCACACCTGCTCTCTTCATGATTGTAAACTATATCATAACCATCCGAGGTGGTGTTGGCTCCCGTAT

ChE – 7F

ATACGGGAGCCAACACCACATAGCCAACCTCAGCCACACCGACTACGCTTGGCCAGAGCAGGTGTGACGGAT

ChE – 7R

ATCCGTCACACCTGCTCTGGCCAAGCGTAGTCGGTGTGGCTGAGGTTGGCTATGTGGTGTTGGCTCCCGTAT

ChE – 8F

ATACGGGAGCCAACACCATTTATCTAATGGCAACTAGGGATAGTGAAAACTACCAGAGCAGGTGTGACGGAT

ChE – 8R

ATCCGTCACACCTGCTCTCCTCCTAGGAATGTAGTCGGGCGGATAAAGTGACAATGGTGTTGGCTCCCGTAT

ChE – 9F

ATACGGGAGCCAACACCATTGTCACTTTATCCGCCCGACTACATTCCTAGGAGGAGAGCAGGTGTGACGGAT

ChE – 9R

ATCCGTCACACCTGCTCTCCTCCTAGGAATGTAGTCGGGCGGATAAAGTGACAATGGTGTTGGCTCCCGTAT

ChE – 10F

ATACGGGAGCCAACACCACCTTCGACGCCAACGACGAACGGCTTTGAAAGGCTAAGAGCAGGTGTGACGGAT

ChE – 10R

ATCCGTCACACCTGCTCTTAGCCTTTCAAAGCCGTTCGTCGTTGGCGTCGAAGGTGGTGTTGGCTCCCGTAT

ChE – 11F

ATACGGGAGCCAACACCAATGGAAGCCGTACCTTCACACCCGTTATTTAAAAACAGAGCAGGTGTGACGGAT

ChE – 11R

ATCCGTCACACCTGCTCTGTTTTTAAATAACGGGTGTGAAGGTACGGCTTCCATTGGTGTTGGCTCCCGTAT

ChE – 12F

ATACGGGAGCCAACACCACGCTGGCCGGGAGGCCCGTCCAAGCCATTACCGTCAAGAGCAGGTGTGACGGAT

ChE – 12R

ATCCGTCACACCTGCTCTTGACGGTAATGGCTTGGACGGGCCTCCCGGCCAGCGTGGTGTTGGCTCCCGTAT

ChE – 13F

ATACGGGAGCCAACACCACATATCACCGCACGCCTATTCCATGTGACGAATCTAAGAGCAGGTGTGACGGAT

ChE – 13R

ATCCGTCACACCTGCTCTTAGATTCGTCACATGGAATAGGCGTGCGGTGATATGTGGTGTTGGCTCCCGTAT

ChE – 15F

ATACGGGAGCCAACACCACGGCGGGCGGGGCATCTCGTGGGGGACGAAGGCGCAAGAGCAGGTGTGACGGAT

ChE – 15R

ATCCGTCACACCTGCTCTTGCGCCTTCGTCCCCCACGAGATGCCCCGCCCGCCGTGGTGTTGGCTCCCGTAT

ChE – 16F

ATACGGGAGCCAACACCATCCTCCTGCGACGTCTGGAGAACAGCCTCTACTTTAAGAGCAGGTGTGACGGAT

ChE – 16R

ATCCGTCACACCTGCTCTTAAAGTAGAGGCTGTTCTCCAGACGTCGCAGGAGGATGGTGTTGGCTCCCGTAT

ChE – 17F

ATACGGGAGCCAACACCAA***AACCCGCA***CTACATCTCCTCTGCCCCCTTCTGATAAGAGCAGGTGTGACGGAT

ChE – 17R

ATCCGTCACACCTGCTCTTATCAGAAGGGGGCAGAGGAGATGTAGTGCGGGTTTTGGTGTTGGCTCCCGTAT

ChE – 18F

ATACGGGAGCCAACACCAGTGGTCTTGTTTTGGATGTTTAGTGATGCGGGTTCTAGAGCAGGTGTGACGGAT

ChE – 18R

ATCCGTCACACCTGCTCTAG***AACCCGCA***TCACTAAACATCCAAAACAAGACCACTGGTGTTGGCTCCCGTAT

ChE – 19F

ATACGGGAGCCAACACCATCTTTCGTGATAGCTATTAAGGCCTATTCGTATCGTAGAGCAGGTGTGACGGAT

ChE -19R

ATCCGTCACACCTGCTCTACGATACGAATAGGCCTTAATAGCTATCACGAAAGATGGTGTTGGCTCCCGTAT

ChE – 20F

ATACGGGAGCCAACACCATACTGAAGCCATACGTCTGTCCAACCGTCATAACTTAGAGCAGGTGTGACGGAT

ChE – 20R

ATCCGTCACACCTGCTCTAAGTTATGACGGTTGGACAGACGTATGGCTTCAGTATGGTGTTGGCTCCCGTAT

**Crimean-Congo (CCHF) Altamura Gn611**

Gn6 - 1F

ATACGGGAGCCAACACCAACTGAAAACTAAGACTTGGTTCCAAATCCTTTCTCTAGAGCAGGTGTGACGGAT

Gn6 - 1R

ATCCGTCACACCTGCTCTAGAGAAAGGATTTGGAACCAAGTCTTAGTTTTCAGTTGGTGTTGGCTCCCGTAT

Gn6 - 2F

ATACGGGAGCCAACACCAGGCCGGAGACTAGCCGAACCCTACTTTTTACTGTGTAGAGCAGGTGTGACGGAT

Gn6 - 2R

ATCCGTCACACCTGCTCTACACAGTAAAAAGTAGGGTTCGGCTAGTCTCCGGCCTGGTGTTGGCTCCCGTAT

Gn6 - 4F

ATACGGGAGCCAACACCACGTGCTGACTATACTATTCAAAAACAACACCCTAGGAGAGCAGGTGTGACGGAT

Gn6 - 4R

ATCCGTCACACCTGCTCTCCTAGGGTGTTGTTTTTGAATAGTATAGTCAGCACGTGGTGTTGGCTCCCGTAT

Gn6 - 5F

ATACGGGAGCCAACACCACGCCTTGTCTATTCTCTTAGTTTCCTGCTACTCCACAGAGCAGGTGTGACGGAT

Gn6 - 5R

ATCCGTCACACCTGCTCTGTGGAGTAGCAGGAAACTAAGAGAATAGACAAGGCGTGGTGTTGGCTCCCGTAT

Gn6 - 6F

ATACGGGAGCCAACACCAGGATAGTTACCAGTCCCTTGTTAAAAATTTATATGCAGAGCAGGTGTGACGGAT

Gn6 - 6R

ATCCGTCACACCTGCTCTGCATATAAATTTTTAACAAGGGACTGGTAACTATCCTGGTGTTGGCTCCCGTAT

Gn6 - 7aF

ATACGGGAGCCAACACCATAGCTTTAGGTTACTTTTCAGACACTATATGTCCCAGAGCAGGTGTGACGGAT

Gn6 - 7aR

ATCCGTCACACCTGCTCTGGGACATATAGTGTCTGAAAAGTAACCTAAAGCTATGGTGTTGGCTCCCGTAT

Gn6 - 7bF

ATACGGGAGCCAACACCAATGCCCGCCTCGATAGAGACTGACCAGTATGTGAGAGCAGGTGTGACGGAT

Gn6 - 7bR

ATCCGTCACACCTGCTCTCACATACTGGTCAGTCTCTATCGAGGCGGGCATTGGTGTTGGCTCCCGTAT

Gn6 - 7cF

ATACGGGAGCCAACACCACCTCATAGTTATGTAATAACGCTTATCTTGTCCGGCAGAGCAGGTGTGACGGAT

Gn6 - 7cR

ATCCGTCACACCTGCTCTGCCGGACAAGATAAGCGTTATTACATAACTATGAGGTGGTGTTGGCTCCCGTAT

Gn6 - 8F

ATACGGGAGCCAACACCACCCATCTCAACCACCGTACCTCACTCGGCGACTTACAGAGCAGGTGTGACGGAT

Gn6 - 8R

ATCCGTCACACCTGCTCTGTAAGTCGCCGAGTGAGGTACGGTGGTTGAGATGGGTGGTGTTGGCTCCCGTAT

Gn6 - 9F

ATACGGGAGCCAACACCACGCTGTCCCCAAGACATTCAGTCTTTGCAACCCGGTAGAGCAGGTGTGACGGAT

Gn6 - 9R

ATCCGTCACACCTGCTCTACCGGGTTGCAAAGACTGAATGTCTTGGGGACAGCGTGGTGTTGGCTCCCGTAT

Gn6 - 10F

ATACGGGAGCCAACACCACGCCATCCCCTTGACACTACCACTAAATCGGCGGTCAGAGCAGGTGTGACGGAT

Gn6 - 10R

ATCCGTCACACCTGCTCTGACCGCCGATTTAGTGGTAGTGTCAAGGGGATGGCGTGGTGTTGGCTCCCGTAT

Gn6 - 11F

ATACGGGAGCCAACACCAATAGATGGATAAGGGGGAAACTGCCATTCGGTTAGTAGAGCAGGTGTGACGGAT

Gn6 - 11R

ATCCGTCACACCTGCTCTACTAACCGAATGGCAGTTTCCCCCTTATCCATCTATTGGTGTTGGCTCCCGTAT

Gn6 - 12F

ATACGGGAGCCAACACCATTACCAGGACTAACTCGTTTTGCACTGGTCTCAGTCAGAGCAGGTGTGACGGAT

Gn6 - 12R

ATCCGTCACACCTGCTCTGACTGAGACCAGTGCAAAACGAGTTAGTCCTGGTAATGGTGTTGGCTCCCGTAT

Gn6 - 13F

ATACGGGAGCCAACACCACGGACGCGTACAGAGTTTATTCCTGAGATCCGTGCTAGAGCAGGTGTGACGGAT

Gn6 - 13R

ATCCGTCACACCTGCTCTAGCACGGATCTCAGGAATAAACTCTGTACGCGTCCGTGGTGTTGGCTCCCGTAT

Gn6 - 14F

ATACGGGAGCCAACACCAGAAAAAAACAAACCCAAGGAATTACACCACAAAAGTAGAGCAGGTGTGACGGAT

Gn6 - 14R

ATCCGTCACACCTGCTCTACTTTTGTGGTGTAATTCCTTGGGTTTGTTTTTTTCTGGTGTTGGCTCCCGTAT

Gn6 - 15F

ATACGGGAGCCAACACCACATGTATTACACAGCTCGCATCTTCTTACCTGGCCCAGAGCAGGTGTGACGGAT

Gn6 - 15R

ATCCGTCACACCTGCTCTGGGCCAGGTAAGAAGATGCGAGCTGTGTAATACATGTGGTGTTGGCTCCCGTAT

Gn6 - 16aF

ATACGGGAGCCAACACCAGCCTTTCCACCTACACTAGCTATCTTATCTCCTTATAGAGCAGGTGTGACGGAT

Gn6 - 16aR

ATCCGTCACACCTGCTCTATAAGGAGATAAGATAGCTAGTGTAGGTGGAAAGGCTGGTGTTGGCTCCCGTAT

Gn6 - 16bF

ATACGGGAGCCAACACCATTAGGTTGGAATTTACATTCATGTTCTGTGGTCATAAGAGCAGGTGTGACGGAT

Gn6 - 16bR

ATCCGTCACACCTGCTCTTATGACCACAGAACATGAATGTAAATTCCAACCTAATGGTGTTGGCTCCCGTAT

Gn6 - 16cF

ATACGGGAGCCAACACCAGAGCACACTAATCATGGCGGCCCGGCGCATCCCGACAGAGCAGGTGTGACGGAT

Gn6 - 16cR

ATCCGTCACACCTGCTCTGTCGGGATGCGCCGGGCCGCCATGATTAGTGTGCTCTGGTGTTGGCTCCCGTAT

Gn6 - 17F (71 bases)

ATACGGGAGCCAACACCAAACTAGACAACCGCCCTTATACACACTGTACCAGTAGAGCAGGTGTGACGGAT

Gn6 - 17R (71 bases)

ATCCGTCACACCTGCTCTACTGGTACAGTGTGTATAAGGGCGGTTGTCTAGTTTGGTGTTGGCTCCCGTAT

Gn6 - 18F

ATACGGGAGCCAACACCATACGCACCCGCATTCGTTAGTGTGTACAGTATGTCCAGAGCAGGTGTGACGGAT

Gn6 - 18R

ATCCGTCACACCTGCTCTGGACATACTGTACACACTAACGAATGCGGGTGCGTATGGTGTTGGCTCCCGTAT

Gn6 - 19F

ATACGGGAGCCAACACCAGCATCCCCGAATAAATAATGCTGCGCTGTTAAAGATAGAGCAGGTGTGACGGAT

Gn6 - 19R

ATCCGTCACACCTGCTCTATCTTTAACAGCGCAGCATTATTTATTCGGGGATGCTGGTGTTGGCTCCCGTAT

Gn6 - 20F (70 bases)

ATACGGGAGCCAACACCAAATTCCTCGTTGACCCCTAACTGTACTCTTAGCCAGAGCAGGTGTGACGGAT

Gn6 - 20R (70 bases)

ATCCGTCACACCTGCTCTGGCTAAGAGTACAGTTAGGGGTCAACGAGGAATTTGGTGTTGGCTCCCGTAT

Gn6 - 21F

ATACGGGAGCCAACACCACCCATTCTGAGACCCCCCGCGCATGTATTGGTCTTGAGAGCAGGTGTGACGGAT

Gn6 - 21R

ATCCGTCACACCTGCTCTCAAGACCAATACATGCGCGGGGGGTCTCAGAATGGGTGGTGTTGGCTCCCGTAT

Gn6 - 22F

ATACGGGAGCCAACACCATGCTAGTGCCCCCACAGACGCACACTAAAGTATTCCAGAGCAGGTGTGACGGAT

Gn6 - 22R

ATCCGTCACACCTGCTCTGGAATACTTTAGTGTGCGTCTGTGGGGGCACTAGCATGGTGTTGGCTCCCGTAT

Gn6 - 23F (71 bases)

ATACGGGAGCCAACACCAGGCCGTGCGCGCTCATTTTGAGAACCACTGCCCCCAGAGCAGGTGTGACGGAT

Gn6 - 23R (71 bases)

ATCCGTCACACCTGCTCTGGGGGCAGTGGTTCTCAAAATGAGCGCGCACGGCCTGGTGTTGGCTCCCGTAT

Gn6 - 24F

ATACGGGAGCCAACACCAGTACTACCCACGGGCTTATTACCCCCTCATCCTTGCAGAGCAGGTGTGACGGAT

Gn6 - 24R

ATCCGTCACACCTGCTCTGCAAGGATGAGGGGGTAATAAGCCCGTGGGTAGTACTGGTGTTGGCTCCCGTAT

Gn6 - 25F

ATACGGGAGCCAACACCATTATGTTAACAAAGGCATACGGCAAGCTCTAACTGTAGAGCAGGTGTGACGGAT

Gn6 - 25R

ATCCGTCACACCTGCTCTACAGTTAGAGCTTGCCGTATGCCTTTGTTAACATAATGGTGTTGGCTCCCGTAT

Gn6 - 27F

ATACGGGAGCCAACACCACCTCAAGATAGCCGTTCATCCGACTGTCGCCATTGTAGAGCAGGTGTGACGGAT

Gn6 - 27R

ATCCGTCACACCTGCTCTACAATGGCGACAGTCGGATGAACGGCTATCTTGAGGTGGTGTTGGCTCCCGTAT

Gn6 - 28F

ATACGGGAGCCAACACCACATAATGGACAATCCCACTGGGCACGTTCTATAACCAGAGCAGGTGTGACGGAT

Gn6 - 28R

ATCCGTCACACCTGCTCTGGTTATAGAACGTGCCCAGTGGGATTGTCCATTATGTGGTGTTGGCTCCCGTAT

Gn6 - 29F

ATACGGGAGCCAACACCAAGCCCGAGCCCGCCGTTATATCCCATCGAGTTCCCCAGAGCAGGTGTGACGGAT

Gn6 - 29R

ATCCGTCACACCTGCTCTGGGGAACTCGATGGGATATAACGGCGGGCTCGGGCTTGGTGTTGGCTCCCGTAT

Gn6 - 30F (71 bases)

ATACGGGAGCCAACACCATCCCACCGAATATCCGCTTTCCTCGTCCTCCTTTCAGAGCAGGTGTGACGGAT

Gn6 - 30R (71 bases)

ATCCGTCACACCTGCTCTGAAAGGAGGACGAGGAAAGCGGATATTCGGTGGGATGGTGTTGGCTCCCGTAT

**CCHF 11E7a Peptide**

E7A -1 F

ATACGGGAGCCAACACCAGCCTCGCCTTCAGATGTTCACTGCTGTTTATTGCATAGAGCAGGTGTGACGGAT

E7A -1 R

ATCCGTCACACCTGCTCTATGCAATAAACAGCAGTGAACATCTGAAGGCGAGGCTGGTGTTGGCTCCCGTAT

**E7A - 2/6/28/30b F**

ATACGGGAGCCAACACCAATAGATGGATAAGGGGGAAACTGCCATTCGGTTAGTAGAGCAGGTGTGACGGAT

**E7A - 2/6/28/30b R**

ATCCGTCACACCTGCTCTACTAACCGAATGGCAGTTTCCCCCTTATCCATCTATTGGTGTTGGCTCCCGTAT

**E7A – 3/10/16/19/21/22 F**

ATACGGGAGCCAACACCAGTCCGTTATGACATGTCCGGACCCGTACGCGTGTCAAGAGCAGGTGTGACGGAT

**E7A – 3/10/16/19/21/22 R**

ATCCGTCACACCTGCTCTTGACACGCGT**ACGGGTCCGGACATGTC**ATAACGGACTGGTGTTGGCTCCCGTAT

E7A - 4 F (71 bases)

ATACGGGAGCCAACACCAGGACTCGCGCAAATAATTTTTATACGCACCACTTCAGAGCAGGTGTGACGGAT

E7A - 4 R (71 bases)

ATCCGTCACACCTGCTCTGAAGTGGTGCGTATAAAAATTATTTGCGCGAGTCCTGGTGTTGGCTCCCGTAT

E7A - 5 F (73 bases)

ATACGGGAGCCAACACCAGTCCGTTATGACATGTCCGGACCCCGTACGCGTGTCAAGAGCAGGTGTGACGGAT

E7A - 5 R (73 bases)

ATCCGTCACACCTGCTCTTGACACGCGTACGGGGTCCGGACATGTCATAACGGACTGGTGTTGGCTCCCGTAT

E7A - 7 F

ATACGGGAGCCAACACCATAAAGCTCGTATTGCCACCCCCCTGTTATTTAATACAGAGCAGGTGTGACGGAT

E7A - 7 R

ATCCGTCACACCTGCTCTGTATTAAATAACAGGGGGGTGGCAATACGAGCTTTATGGTGTTGGCTCCCGTAT

E7A - 8 F (71 bases)

ATACGGGAGCCAACACCATCACGGCAATGTCCCGATAATGTCTTGCTTCAGCGAGAGCAGGTGTGACGGAT

E7A - 8 R (71 bases)

ATCCGTCACACCTGCTCTCGCTGAAGCAAGACATTATCGGGACATTGCCGTGATGGTGTTGGCTCCCGTAT

E7A - 11 F (71 bases)

ATACGGGAGCCAACACCAGCAGTACTAACCCCCCTTACCATATATATCACACGAGAGCAGGTGTGACGGAT

E7A - 11 R (71 bases)

ATCCGTCACACCTGCTCTCGTGTGATATATATGGTAAGGGGGGTTAGTACTGCTGGTGTTGGCTCCCGTAT

**E7A - 12/30a/32/34 F**

ATACGGGAGCCAACACCACCCTAAATTCCAGAGTGTACAAGAGAACGAACTACCAGAGCAGGTGTGACGGAT

**E7A - 12/30a/32/34 R**

ATCCGTCACACCTGCTCTGGTAGTTCGTTCTCTTGTACACTCTGGAATTTAGGGTGGTGTTGGCTCCCGTAT

E7A - 13 F (71 bases)

ATACGGGAGCCAACACCATAGCGACTTGGCAAAAAATTTACATCCATTACTCCAGAGCAGGTGTGACGGAT

E7A - 13 R (71 bases)

ATCCGTCACACCTGCTCTGGAGTAATGGATGTAAATTTTTTGCCAAGTCGCTATGGTGTTGGCTCCCGTAT

E7A - 14 F (71 bases)

ATACGGGAGCCAACACCACGTACACAAACCAAATACGCACCTTCCCACCCTCCAGAGCAGGTGTGACGGAT

E7A - 14 R (71 bases)

ATCCGTCACACCTGCTCTGGAGGGTGGGAAGGTGCGTATTTGGTTTGTGTACGTGGTGTTGGCTCCCGTAT

E7A - 15 F (59 bases)

ATACGGGAGCCAACACCACATCTAGCACGAGACCCTATCCCAGAGCAGGTGTGACGGAT

E7A - 15 R (59 bases)

ATCCGTCACACCTGCTCTGGGATAGGGTCTCGTGCTAGATGTGGTGTTGGCTCCCGTAT

E7A - 17 F

ATACGGGAGCCAACACCAAATCGTCAACAGCCCTGCGCCACTTATCTTTTTGCCAGAGCAGGTGTGACGGAT

E7A - 17 R

ATCCGTCACACCTGCTCTGGCAAAAAGATAAGTGGCGCAGGGCTGTTGACGATTTGGTGTTGGCTCCCGTAT

E7A - 18 F (73 bases)

ATACGGGAGCCAACACCAACAGATGGATAAGGGGGAAACTGCCCATTCGGTTAGTAGAGCAGGTGTGACGGAT

E7A - 18 R (73 bases)

ATCCGTCACACCTGCTCTACTAACCGAATGGGCAGTTTCCCCCTTATCCATCTGTTGGTGTTGGCTCCCGTAT

E7A - 20 F (71 bases)

ATACGGGAGCCAACACCACGCAGTTATAACGGCAGGCCCCATATCGTTTAACCAGAGCAGGTGTGACGGAT

E7A - 20 R (71 bases)

ATCCGTCACACCTGCTCTGGTTAAACGATATGGGGCCTGCCGTTATAACTGCGTGGTGTTGGCTCCCGTAT

E7A - 23 F (67 bases)

ATACGGGAGCCAACACCATGTCAGGACCTCCATCGCCCGGGCCCGCCGCCGCTGAGAGCAGGTGTGAC

E7A - 23 R (67 bases)

GTCACACCTGCTCTCAGCGGCGGCGGGCCCGGGCGATGGAGGTCCTGACATGGTGTTGGCTCCCGTAT

E7A - 25 F

ATACGGGAGCCAACACCATAACAAATAACCACCCTCAATGCTAGATAGTGGCAGAGCAGGTGTGACGGAT

E7A - 25 R

ATCCGTCACACCTGCTCTGCCACTATCTAGCATTGAGGGTGGTTATTTGTTATGGTGTTGGCTCCCGTAT

E7A - 29 F

ATACGGCAGCCAACACCACACACATAGCGCTTTGTATTCAGCCGGATGTGATGTAGAGCAGGTGTGACGGAT

E7A - 29 R

ATCCGTCACACCTGCTCTACATCACATCCGGCTGAATACAAAGCGCTATGTGTGTGGTGTTGGCTGCCGTAA

E7A - 33 F

ATACGGGAGCCAACACCACACACAGAGCGCCATGGACTCAGTCAGATGTGATGTAGAGCAGGTGTGACGGAT

E7A - 33 R

ATCCGTCACACCTGCTCTACATCACATCTGACTGAGTCCATGGCGCTCTGTGTGTGGTGTTGGCTCCCGTAT

E7A - 35 F

ATACGGGAGCCAACGCCACAGGCGTGACATCACCCGTACCCTACCTTAGTGCCAGAGCAGGTGTGACGGAT

E7A - 35 R

ATCCGTCACACCTGCTCTGGCACTAAGGTAGGGTACGGGTGATGTCACGCCTGTGGCGTTGGCTCCCGTAT

**CCHF 11E7b Peptide**

E7B -1a F

ATACGGGAGCCAACACCATAATTCAAGAGGATTCCTCAAAATATGAAGCTTCCAGAGCAGGTGTGACGGAT

E7B -1a R

ATCCGTCACACCTGCTCTGGAAGCTTCATATTTTGAGGAATCCTCTTGAATTATGGTGTTGGCTCCCGTAT

E7B – 1bF

ATACGGGAGCCAACACCAGTCCGTTATGACATGTCCGGACCCGTACGCGTGTCAAAGAGCAGGTGTGACGGAT

E7B – 1bR

ATCCGTCACACCTGCTCTTTGACACGCGT**ACGGGTCCGGACATGTC**ATAACGGACTGGTGTTGGCTCCCGTAT

E7B - 3 F

ATACGGGAGCCAACACCATATCTTATCATAATGTGATGCTAAGAAGGATCCTTTAGAGCAGGTGTGACGGAT

E7B - 3 R

ATCCGTCACACCTGCTCTAAAGGATCCTTCTTAGCATCACATTATGATAAGATATGGTGTTGGCTCCCGTAT

E7B - 4 F

ATACGGGAGCCAACACCAGATTGATGTAAGTAGCCCTCAAATGATTTAAAGTTTAGAGCAGGTGTGACGGAT

E7B - 4 R

ATCCGTCACACCTGCTCTAAACTTTAAATCATTTGAGGGCTACTTACATCAATCTGGTGTTGGCTCCCGTAT

E7B - 5 F

ATACGGGAGCCAACACCACACTAATTTATCGCATGCATCGCCCGCTGATGCCCAAGAGCAGGTGTGACGGAT

E7B - 5 R

ATCCGTCACACCTGCTCTTGGGCATCAGCGGGCGATGCATGCGATAAATTAGTGTGGTGTTGGCTCCCGTAT

E7B - 6 F

ATACGGGAGCCAACACCACAGAAGTTATTTTGAGAACGCGACCCAAATAGGTTAAGAGCAGGTGTGACGGAT

E7B - 6 R

ATCCGTCACACCTGCTCTTAACCTATTTGGGTCGCGTTCTCAAAATAACTTCTGTGGTGTTGGCTCCCGTAT

E7B - 7 F

ATACGGGAGCCAACACCATTAATGTAGAACACCACTCTTATTGACAAGCCTATTAGAGCAGGTGTGACGGAT

E7B - 7 R

ATCCGTCACACCTGCTCTAATAGGCTTGTCAATAAGAGTGGTGTTCTACATTAATGGTGTTGGCTCCCGTAT

**E7B – 8a/10/16-19/23-25 F**

ATACGGGAGCCAACACCAGTCCGTTATGACATGTCCGGACCCGTACGCGTGTCAAGAGCAGGTGTGACGGAT

**E7B – 8a/10/16-19/23-25 R**

ATCCGTCACACCTGCTCTTGACACGCGT**ACGGGTCCGGACATGTC**ATAACGGACTGGTGTTGGCTCCCGTAT

E7B – 8b F

ATACGGGAGCCAACACCACAGTTTGTAGTGTAACAATGCTAGATAATAATGAAAAGAGCAGGTGTGACGGAT

E7B – 8b R

ATCCGTCACACCTGCTCTTTTCATTATTATCTAGCATTGTTACACTACAAACTGTGGTGTTGGCTCCCGTAT

E7B - 11 F

ATACGGGAGCCAACACCAAGCAAATCACCAGAAATCTTTTAACAATCTATTGACAGAGCAGGTGTGACGGAT

E7B - 11 R

ATCCGTCACACCTGCTCTGTCAATAGATTGTTAAAAGATTTCTGGTGATTTGCTTGGTGTTGGCTCCCGTAT

E7B - 12 F

ATACGGGAGCCAACACCACACAGGAACTAGAAGAAAGTATCTTTTTTCGATTTAAGAGCAGGTGTGACGGAT

E7B - 12 R

ATCCGTCACACCTGCTCTTAAATCGAAAAAAGATACTTTCTTCTAGTTCCTGTGTGGTGTTGGCTCCCGTAT

E7B - 13/15 F (59 bases)

ATACGGGAGCCAACACCACATCTAGCACGAGACCCTATCCCAGAGCAGGTGTGACGGAT

E7B - 13/15 R (59 bases)

ATCCGTCACACCTGCTCTGGGATAGGGTCTCGTGCTAGATGTGGTGTTGGCTCCCGTAT

E7B - 14 F

TTACGGGAGCCAGCACCATTCCGTTATGACGTGTCCGGACCCGTTCGCGCGTCAAGAGCAGGTGTGACGGAT

E7B - 14 R

ATCCGTCACACCTGCTCTTGACGCGCGA**ACGGGTCCGGACA**CGTCATAACGGAATGGTGCTGGCTCCCGTAA

E7B - 20/21 F (71 bases)

ATACGGGAGCCAACACCATCACGGCAATGTCCCGATAATGTCTTGCTTCAGCGAGAGCAGGTGTGACGGAT

E7B - 20/21 R (71 bases)

ATCCGTCACACCTGCTCTCGCTGAAGCAAGACATTATCGGGACATTGCCGTGATGGTGTTGGCTCCCGTAT

E7B - 22 F (55 bases)

ATACGGGAGCCAACACCAGTCCGTTATGACATTGTCAAGAGCAGGTGTGACGGAT

E7B - 22 R (55 bases)

ATCCGTCACACCTGCTCTTGACAATGTCATAACGGACTGGTGTTGGCTCCCGTAT

E7B - 26 F (71 bases)

ATACGGGAGCCAACACCACATACTCAGACGATTACCCAGCGCATGCTTGTAACAGAGCAGGTGTGACGGAT

E7B - 26 R (71 bases)

ATCCGTCACACCTGCTCTGTTACAAGCATGCGCTGGGTAATCGTCTGAGTATGTGGTGTTGGCTCCCGTAT

E7B - 27 F (71 bases)

ATACGGGAGCCAACACCACTCTCTAGCCCACGGCGGGGTTTTCTCGCAAGTCCAGAGCAGGTGTGACGGAT

E7B - 27 R (71 bases)

ATCCGTCACACCTGCTCTGGACTTGCGAGAAAACCCCGCCGTGGGCTAGAGAGTGGTGTTGGCTCCCGTAT

E7B - 30 F (71 bases)

ATACGGGAGCCAACACCAATTGCGCCCTAAGGCTACCCACATTACCCATGTGTAGAGCAGGTGTGACGGAT

E7B - 30 R (71 bases)

ATCCGTCACACCTGCTCTACACATGGGTAATGTGGGTAGCCTTAGGGCGCAATTGGTGTTGGCTCCCGTAT

E7B - 31 F (71 bases)

ATACGGGAGCCAACACCAGCTGCTGCTTCAACGAAATCCCAGGCACCCTGACAAGAGCAGGTGTGACGGAT

E7B - 31 R (71 bases)

ATCCGTCACACCTGCTCTTGTCAGGGTGCCTGGGATTTCGTTGAAGCAGCAGCTGGTGTTGGCTCCCGTAT

E7B - 33 F (71 bases)

ATACGGGAGCCAACACCAGTACCTGATACCGGGGTACATAAACACCAACATCTAGAGCAGGTGTGACGGAT

E7B - 33 R (71 bases)

ATCCGTCACACCTGCTCTAGATGTTGGTGTTTATGTACCCCGGTATCAGGTACTGGTGTTGGCTCCCGTAT

E7B - 34 F (71 bases)

ATACGGGAGCCAACACCAGATACCGTGAATATACTAATTTCGCAATAGTTAATAGAGCAGGTGTGACGGAT

E7B - 34 R (71 bases)

ATCCGTCACACCTGCTCTATTAACTATTGCGAAATTAGTATATTCACGGTATCTGGTGTTGGCTCCCGTAT

**CCHF 11E7c Peptide**

**E7C - 1a/18/20F**

ATACGGGAGCCAACACCAATAGATGGATAAGGGGGAAACTGCCATTCGGTTAGTAGAGCAGGTGTGACGGAT

**E7C - 1a/18/20R**

ATCCGTCACACCTGCTCTACTAACCGAATGGCAGTTTCCCCCTTATCCATCTATTGGTGTTGGCTCCCGTAT

E7C - 1bF

ATACGGGAGCCAACACCACATCTACGCCCAAGCCTCTATGTACAAGTAGCAACAAGAGCAGGTGTGACGGAT

E7C - 1bR

ATCCGTCACACCTGCTCTTGTTGCTACTTGTACATAGAGGCTTGGGCGTAGATGTGGTGTTGGCTCCCGTAT

E7C - 2F (71)

ATACGGGAGCCAACACCAATCTCCACTGTGAACCTTATCGAGTTTTTTGTACGAGAGCAGGTGTGACGGAT

E7C - 2R (71)

ATCCGTCACACCTGCTCTCGTACAAAAAACTCGATAAGGTTCACAGTGGAGATTGGTGTTGGCTCCCGTAT

E7C - 4aF

ATACGGGAGCCAACACCAGCACGCCCTTTTAGTGTCCAACTGAATCTTCACCTAAGAGCAGGTGTGACGGAT

E7C - 4aR

ATCCGTCACACCTGCTCTTAGGTGAAGATTCAGTTGGACACTAAAAGGGCGTGCTGGTGTTGGCTCCCGTAT

E7C - 4bF

ATACGGGAGCCAACACCATGCTTTTGGAGTATTTCGCCTCCAAGCTACTCCCCTAGAGCAGGTGTGACGGAT

E7C - 4bR

ATCCGTCACACCTGCTCTAGGGGAGTAGCTTGGAGGCGAAATACTCCAAAAGCATGGTGTTGGCTCCCGTAT

E7C - 5F

ATACGGGAGCCAACACCATTGATCCTGCCGGTTCGCCCCTTGTTCCCACCTTTTAGAGCAGGTGTGACGGAT

E7C - 5R

ATCCGTCACACCTGCTCTAAAAGGTGGGAACAAGGGGCGAACCGGCAGGATCAATGGTGTTGGCTCCCGTAT

E7C - 6F

ATACGGGAGCCAACACCACCACTGTTTAGGCACAACTTGCTTTCTTAGCCCCGCAGAGCAGGTGTGACGGAT

E7C - 6R

ATCCGTCACACCTGCTCTGCGGGGCTAAGAAAGCAAGTTGTGCCTAAACAGTGGTGGTGTTGGCTCCCGTAT

E7C - 7aF

ATACGGGAGCCAACACCACGCGTTTATTATGTTCCCCATGATTGCCACGGCTACAGAGCAGGTGTGACGGAT

E7C - 7aR

ATCCGTCACACCTGCTCTGTAGCCGTGGCAATCATGGGGAACATAATAAACGCGTGGTGTTGGCTCCCGTAT

E7C - 7bF

ATACGGGAGCCAACACCATATACTGCCGCAGTTTGGGCCCGCAGTCCATGGGCAAGAGCAGGTGTGACGGAT

E7C - 7bR

ATCCGTCACACCTGCTCTTGCCCATGGACTGCGGGCCCAAACTGCGGCAGTATATGGTGTTGGCTCCCGTAT

E7C - 8F

ATACGGGAGCCAACACCACCTAAGTAATGCCAAAAACAACTCGGGTACGCAATGAGAGCAGGTGTGACGGAT

E7C - 8R

ATCCGTCACACCTGCTCTCATTGCGTACCCGAGTTGTTTTTGGCATTACTTAGGTGGTGTTGGCTCCCGTAT

E7C - 9F

ATACGGGAGCCAACACCACTTCTCTGTGACCAGTATACGTCCCATTTCCCTATTAGAGCAGGTGTGACGGAT

E7C - 9R

ATCCGTCACACCTGCTCTAATAGGGAAATGGGACGTATACTGGTCACAGAGAAGTGGTGTTGGCTCCCGTAT

E7C - 10F

ATACGGGAGCCAACACCAGGATACGTTCCGTGCATGGATGTGCTGCCCCATGTTAGAGCAGGTGTGACGGAT

E7C - 10R

ATCCGTCACACCTGCTCTAACATGGGGCAGCACATCCATGCACGGAACGTATCCTGGTGTTGGCTCCCGTAT

E7C - 11F

ATACGGGAGCCAACACCACCATTTTCGTTTTTCTTGAGTATTTCGACCTTAGTGAGAGCAGGTGTGACGGAT

E7C - 11R

ATCCGTCACACCTGCTCTCACTAAGGTCGAAATACTCAAGAAAAACGAAAATGGTGGTGTTGGCTCCCGTAT

E7C - 12F

ATACGGGAGCCAACACCATTCGAAACCCATAATCTTTTCCTCACTCTGCGTATTAGAGCAGGTGTGACGGAT

E7C - 12R

ATCCGTCACACCTGCTCTAATACGCAGAGTGAGGAAAAGATTATGGGTTTCGAATGGTGTTGGCTCCCGTAT

E7C - 13F

ATACGGGAGCCAACACCACGCATGGGGCTCTCCCTATTACGCAATCCGTTGTAGAGAGCAGGTGTGACGGAT

E7C - 13R

ATCCGTCACACCTGCTCTCTACAACGGATTGCGTAATAGGGAGAGCCCCATGCGTGGTGTTGGCTCCCGTAT

E7C - 14F

ATACGGGAGCCAACACCATCTTGTCCTCGGTCCGTCTTTGCATTCTGGTCTAAAAGAGCAGGTGTGACGGAT

E7C - 14R

ATCCGTCACACCTGCTCTTTTAGACCAGAATGCAAAGACGGACCGAGGACAAGATGGTGTTGGCTCCCGTAT

E7C - 15F

ATACGGGAGCCAACACCACCGCTGTAAGTGCTTGGGTCGACCGCGCCCGCTGCCAGAGCAGGTGTGACGGAT

E7C - 15R

ATCCGTCACACCTGCTCTGGCAGCGGGCGCGGTCGACCCAAGCACTTACAGCGGTGGTGTTGGCTCCCGTAT

E7C - 17F

ATACGGGAGCCAACACCAGGTGACGCAGGTGAGTCTGCCTCCCCATGTGCTCCCAGAGCAGGTGTGACGGAT

E7C - 17R

ATCCGTCACACCTGCTCTGGGAGCACATGGGGAGGCAGACTCACCTGCGTCACCTGGTGTTGGCTCCCGTAT

E7C - 19F

ATACGGGAGCCAACACCACTTCCGGGCTATACCGGGGCTCGCGCAATTCTGACCAGAGCAGGTGTGACGGAT

E7C - 19R

ATCCGTCACACCTGCTCTGGTCAGAATTGCGCGAGCCCCGGTATAGCCCGGAAGTGGTGTTGGCTCCCGTAT

E7C - 21F

ATACGGGAGCCAACACCAGTCTTTTATTCATCATGATCGCTGACCTACACCCCAAGAGCAGGTGTGACGGAT

E7C - 21R

ATCCGTCACACCTGCTCTTGGGGTGTAGGTCAGCGATCATGATGAATAAAAGACTGGTGTTGGCTCCCGTAT

E7C - 22F

ATACGGGAGCCAACACCACTTCAAAAGTCAGATACAAAGACAGAGATTGGACTTAGAGCAGGTGTGACGGAT

E7C - 22R

ATCCGTCACACCTGCTCTAAGTCCAATCTCTGTCTTTGTATCTGACTTTTGAAGTGGTGTTGGCTCCCGTAT

**E7C - 23/25F**

ATACGGGAGCCAACACCATTATGTTAACAAAGGCATACGGCAAGCTCTAACTGTAGAGCAGGTGTGACGGAT

**E7C - 23/25R**

ATCCGTCACACCTGCTCTACAGTTAGAGCTTGCCGTATGCCTTTGTTAACATAATGGTGTTGGCTCCCGTAT

E7C - 24F

ATACGGGAGCCAACACCATAATATTACAATGCCAGAATCTACACATAATCCTATAGAGCAGGTGTGACGGAT

E7C - 24R

ATCCGTCACACCTGCTCTATAGGATTATGTGTAGATTCTGGCATTGTAATATTATGGTGTTGGCTCCCGTAT

E7C - 26F

ATACGGGAGCCAACACCACTTGACGCCGTGGCAACACGCTGACGAGCTTTACCCAGAGCAGGTGTGACGGAT

E7C - 26R

ATCCGTCACACCTGCTCTGGGTAAAGCTCGTCAGCGTGTTGCCACGGCGTCAAGTGGTGTTGGCTCCCGTAT

E7C - 27F

ATACGGGAGCCAACACCAGCCAACTCACTATTACTTAGTAACCCTAACGATGGCAGAGCAGGTGTGACGGAT

E7C - 27R

ATCCGTCACACCTGCTCTGCCATCGTTAGGGTTACTAAGTAATAGTGAGTTGGCTGGTGTTGGCTCCCGTAT

E7C - 28F

ATACGGGAGCCAACACCATGTCCTCTGATCCTCTGCTGATAAGCGGTGGCCACTAGAGCAGGTGTGACGGAT

E7C - 28R

ATCCGTCACACCTGCTCTAGTGGCCACCGCTTATCAGCAGAGGATCAGAGGACATGGTGTTGGCTCCCGTAT

E7C - 29F

ATACGGGAGCCAACACCATACGATCCAATGATGGACCCGTGCGGACTGATTTACAGAGCAGGTGTGACGGAT

E7C - 29F

ATCCGTCACACCTGCTCTGTAAATCAGTCCGCACGGGTCCATCATTGGATCGTATGGTGTTGGCTCCCGTAT

E7C - 30F

ATACGGGAGCCAACACCATTCCATCTCCATGTAGCTAAAGTCGATACTCCATCCAGAGCAGGTGTGACGGAT

E7C - 30R

ATCCGTCACACCTGCTCTGGATGGAGTATCGACTTTAGCTACATGGAGATGGAATGGTGTTGGCTCCCGTAT

**Intact CCHF Virus IbAr10200 Strain (Aldehyde Fixed)**

**10200 – 2-6/8-23/25/26/28/30/31/33/34 F**

ATACGGGAGCCAACACCAGTCCGTTATGACATGTCCGGACCCGTACGCGTGTCAAGAGCAGGTGTGACGGAT

**10200 – 2-6/8-23/25/26/28/30/31/33/34 R**

ATCCGTCACACCTGCTCTTGACACGCGT**ACGGGTCCGGACA**TGTCATAACGGACTGGTGTTGGCTCCCGTAT

**Intact CCHF Virus Drosdov Strain (Aldehyde Fixed)**

**Drosdov – 4-7/10-12/14/18/20-24 F**

ATACGGGAGCCAACACCAGTCCGTTATGACATGTCCGGACCCGTACGCGTGTCAAGAGCAGGTGTGACGGAT

**Drosdov – 4-7/10-12/14/18/20-24 R**

ATCCGTCACACCTGCTCTTGACACGCGT**ACGGGTCCGGACATGTC**ATAACGGACTGGTGTTGGCTCCCGTAT

Drosdov - 13 F

ATACGGGAGCCAACACCAGTCCGTTTGACATGTCCGGACCCGTACGCGTGTCAAGAGCAGGTGTGACGGAT

Drosdov - 13 R

ATCCGTCACACCTGCTCTTGACACGCGT**ACGGGTCCGGACATGTC**AAACGGACTGGTGTTGGCTCCCGTAT

Drosdov - 16 F

ATACGGGAGCCAACACCATCACGGCAATGTCCCGATAATGTCTTGCTTCAGCGAGAGCAGGTGTGACGGAT

Drosdov - 16 R

ATCCGTCACACCTGCTCTCGCTGAAGCAAGACATTATCGGGACATTGCCGTGATGGTGTTGGCTCCCGTAT

Drosdov - 17 F

ATACGGGAGCCAACACCAGTCCGTTATGACATGTCCGGACCCGTACAAGAGCAGGTGTGACGGAT

Drosdov - 17 R

ATCCGTCACACCTGCTCTTGT**ACGGGTCCGGACATGTC**ATAACGGACTGGTGTTGGCTCCCGTAT

Drosdov - 19 F

ATACGGGAGCCAACACCAAGTCCAAGCCAAACAAGAGCATAACACCAAATCTGGAGAGCAGGTGTGACGGAT

Drosdov - 19 R

ATCCGTCACACCTGCTCTCCAGATTTGGTGTTATGCTCTTGTTTGGCTTGGACTTGGTGTTGGCTCCCGTAT

**Dengue Envelope Serotype 1 (DE1)**

DE1-3F

ATACGGGAGCCAACACCACCCTAAATTCCAGAGTGTACAAGAGAACGAACTACCAGAGCAGGTGTGACGGAT

DE1-3R

ATCCGTCACACCTGCTCTGGTAGTTCGTTCTCTTGTACACTCTGGAATTTAGGGTGGTGTTGGCTCCCGTAT

DE1-4F

ATACGGGAGCCAACACCACTACTCATATACCTTATACTATAAACAATCTGCGCGAGAGCAGGTGTGACGGAT

DE1-4R

ATCCGTCACACCTGCTCTCGCGCAGATTGTTTATAGTATAAGGTATATGAGTAGTGGTGTTGGCTCCCGTAT

DE1-5Fa

ATACGGGAGCCAACACCACATTCGTACTAGCCCCGGTTGCCCGTCGACCGGACAAGAGCAGGTGTGACGGAT

DE1-5Fb

ATACGGGAGCCAACACCAGGAGGGCGCGCCTATTTCGCCAATTCGTCCGCAGCGAGAGCAGGTGTGACGGAT

DE1-5Ra

ATCCGTCACACCTGCTCTTGTCCGGTCGACGGGCAACCGGGGCTAGTACGAATGTGGTGTTGGCTCCCGTAT

DE1-5Rb

ATCCGTCACACCTGCTCTCGCTGCGGACGAATTGGCGAAATAGGCGCGCCCTCCTGGTGTTGGCTCCCGTAT

DE1-6F

ATACGGGAGCCAACACCACACATTATCGGCAACTGGCAAGGCTAAGGTACTGGTAGAGCAGGTGTGACGGAT

DE1-6R

ATCCGTCACACCTGCTCTACCAGTACCTTAGCCTTGCCAGTTGCCGATAATGTGTGGTGTTGGCTCCCGTAT

DE1-8F

ATACGGGAGCCAACACCACTGGCGACCCACTCCCCTGGTACGTCACCACAGCCTAGAGCAGGTGTGACGGAT

DE1-8R

ATCCGTCACACCTGCTCTAGGCTGTGGTGACGTACCAGGGGAGTGGGTCGCCAGTGGTGTTGGCTCCCGTAT

DE1-9F

ATACGGGAGCCAACACCAAGACAACCGAGCTAATAGGCATTTCAACACCTGTCCAGAGCAGGTGTGACGGAT

DE1-9R

ATCCGTCACACCTGCTCTGGACAGGTGTTGAAATGCCTATTAGCTCGGTTGTCTTGGTGTTGGCTCCCGTAT

DE1-10F

ATACGGGAGCCAACACCAGAAGACCATGTGAAGTAAAGACTTCAATTATCAGTCAGAGCAGGTGTGACGGAT

DE1-10R

ATCCGTCACACCTGCTCTGACTGATAATTGAAGTCTTTACTTCACATGGTCTTCTGGTGTTGGCTCCCGTAT

**Dengue Envelope Serotype 2 (DE2)**

DE2-2F

ATACGGGAGCCAACACCACAATAAATCCGTGCGCGTGACGCGTTTCATACAGTCAGAGCAGGTGTGACGGAT

DE2-2R

ATCCGTCACACCTGCTCTGACTGTATGAAACGCGTCACGCGCACGGATTTATTGTGGTGTTGGCTCCCGTAT

DE2-4F

ATACGGGAGCCAACACCACTATTGGCTATACATTCGTTGTGAGAAACGCACCGCAGAGCAGGTGTGACGGAT

DE2-4R

ATCCGTCACACCTGCTCTGCGGTGCGTTTCTCACAACGAATGTATAGCCAATAGTGGTGTTGGCTCCCGTAT

DE2-5Fa

ATACGGGAGCCAACACCATGGGGACCAACTGTCCGGAGAGAGTCCTGTCGAGGGAGAGCAGGTGTGACGGAT

DE2-5Fb

ATACGGGAGCCAACACCACATAGACACAAGATATATCATATATTGCTCGCAGAGCAGGTGTGACGGAT

DE2-5Rb

ATCCGTCACACCTGCTCTCCCTCGACAGGACTCTCTCCGGACAGTTGGTCCCCATGGTGTTGGCTCCCGTAT

DE2-5Rb

ATCCGTCACACCTGCTCTGCGAGCAATATATGATATATCTTGTGTCTATGTGGTGTTGGCTCCCGTAT

DE2-6F (69 bases)

ATACGGGAGCCAACACCATTGTAGCTGACAACTGTTTTACATGAACACTTCAGAGCAGGTGTGACGGAT

DE2-6R (69 bases)

ATCCGTCACACCTGCTCTGAAGTGTTCATGTAAAACAGTTGTCAGCTACAATGGTGTTGGCTCCCGTAT

DE2-7F

ATACGGGAGCCAACACCAGGGTGCCAGCAGATTATAATTGAACAAACCAGCGATAGAGCAGGTGTGACGGAT

DE2-7R

ATCCGTCACACCTGCTCTATCGCTGGTTTGTTCAATTATAATCTGCTGGCACCCTGGTGTTGGCTCCCGTAT

DE2-8F

ATACGGGAGCCAACACCAGCACATAGAAAAAAAATACAACCACATCGATTGACCAGAGCAGGTGTGACGGAT

DE2-8R

ATCCGTCACACCTGCTCTGGTCAATCGATGTGGTTGTATTTTTTTTCTATGTGCTGGTGTTGGCTCCCGTAT

DE2-9F

ATACGGGAGCCAACACCAACCAGGTATTGTCCAAAATGGAAACAAATGAGGAATAGAGCAGGTGTGACGGAT

DE2-9R

ATCCGTCACACCTGCTCTATTCCTCATTTGTTTCCATTTTGGACAATACCTGGTTGGTGTTGGCTCCCGTAT

DE2-10F

ATACGGGAGCCAACACCACACACAAAAGGAATTGTATACTCGCATAAGGCCGCCAGAGCAGGTGTGACGGAT

DE2-10R

ATCCGTCACACCTGCTCTGGCGGCCTTATGCGAGTATACAATTCCTTTTGTGTGTGGTGTTGGCTCCCGTAT

**Dengue EnvelopeSerotype 3 (DE3)**

DE3-1F

ATACGGGAGCCAACACCAGAAACGTGGACTGTGTAGGCAAACCTATTATTTTCTAGAGCAGGTGTGACGGAT

DE3-1R

ATCCGTCACACCTGCTCTAGAAAATAATAGGTTTGCCTACACAGTCCACGTTTCTGGTGTTGGCTCCCGTAT

DE3-2F

ATACGGGAGCCAACACCAGCGCAATTGATGACTACCCTAAGAAATCTATTGGCCAGAGCAGGTGTGACGGAT

DE3-2R

ATCCGTCACACCTGCTCTGGCCAATAGATTTCTTAGGGTAGTCATCAATTGCGCTGGTGTTGGCTCCCGTAT

DE3-3Fa

ATACGGGAGCCAACACCACGGCCGAGGTCCACTACCCCTATGGCTGGCCCTTCCAGAGCAGGTGTGACGGAT

DE3-3Fb

ATACGGGAGCCAACACCAGCCTACGGGTGGATGATCCGCGGTGTTCGAGTGTTAGAGCAGGTGTGACGGAT

DE3-3Ra

ATCCGTCACACCTGCTCTGGAAGGGCCAGCCATAGGGGTAGTGGACCTCGGCCGTGGTGTTGGCTCCCGTAT

DE3-3Rb

ATCCGTCACACCTGCTCTAACACTCGAACACCGCGGATCATCCACCCGTAGGCTGGTGTTGGCTCCCGTAT

DE3-4Fa

ATACGGGAGCCAACACCATGCAGTATCCACCTTCTCTTTTTTCTCACTCCACTGAGAGCAGGTGTGACGGAT

DE3-4Ra

ATCCGTCACACCTGCTCTCAGTGGAGTGAGAAAAAAGAGAAGGTGGATACTGCATGGTGTTGGCTCCCGTAT

DE3-4Fb

ATCCGTCACACCTGCTCTCGAATGGGACAACTTCTCGATATCTACTATGGTTGGTGGTGTTGGCTCCCGTAT

DE3-4Rb

ATACGGGAGCCAACACCACCAACCATAGTAGATATCGAGAAGTTGTCCCATTCGAGAGCAGGTGTGACGGAT

DE3-6F

ATACGGGAGCCAACACCAGGGATGGGTAAAGAAAGTCGCGAGACGATGATGCCAGAGCAGGTGTGACGGAT

DE3-6R

ATCCGTCACACCTGCTCTGGCATCATCGTCTCGCGACTTTCTTTACCCATCCCTGGTGTTGGCTCCCGTAT

DE3-9F

ATACGGGAGCCAACACCACGACATCCGTTCTGAACACACGATAGTGATGATTGTAGAGCAGGTGTGACGGAT

DE3-9R

ATCCGTCACACCTGCTCTACAATCATCACTATCGTGTGTTCAGAACGGATGTCGTGGTGTTGGCTCCCGTAT

DE3-10F

ATACGGGAGCCAACACCACGGTATTGTAAAGAAATGAAATCAGTAATATATTCCAGAGCAGGTGTGACGGAT

DE3-10R

ATCCGTCACACCTGCTCTGGAATATATTACTGATTTCATTTCTTTACAATACCGTGGTGTTGGCTCCCGTAT

**Dengue Envelope Serotype 4 (DE4)**

DE4-2F

ATACGGGAGCCAACACCATACAAAAATCCGAAGTTAAGACAGCTCACGCTTATCAGAGCAGGTGTGACGGAT

DE4-2R

ATCCGTCACACCTGCTCTGATAAGCGTGAGCTGTCTTAACTTCGGATTTTTGTATGGTGTTGGCTCCCGTAT

DE4-3F

ATACGGGAGCCAACACCATACATGGCAGCTCCTACAGATCACCACTCTAAGAGTAGAGCAGGTGTGACGGAT

DE4-3R

ATCCGTCACACCTGCTCTACTCTTAGAGTGGTGATCTGTAGGAGCTGCCATGTATGGTGTTGGCTCCCGTAT

DE4-4F

ATACGGGAGCCAACACCACAACTCACCAGGACACTCGGCCGCCCGGTCCCCAATAGAGCAGGTGTGACGGAT

DE4-4R

ATCCGTCACACCTGCTCTATTGGGGACCGGGCGGCCGAGTGTCCTGGTGAGTTGTGGTGTTGGCTCCCGTAT

DE4-5F

ATACGGGAGCCAACACCAGTTGACAACACATGACTCTACACGATATGTCACACAAGAGCAGGTGTGACGGAT

DE4-5R

ATCCGTCACACCTGCTCTTGTGTGACATATCGTGTAGAGTCATGTGTTGTCAACTGGTGTTGGCTCCCGTAT

DE4-6F

ATACGGGAGCCAACACCAGGCTATGAAGAAAGAAAAATGAGTAACACATAACGCAGAGCAGGTGTGACGGAT

DE4-6R

ATCCGTCACACCTGCTCTGCGTTATGTGTTACTCATTTTTCTTTCTTCATAGCCTGGTGTTGGCTCCCGTAT

DE4-7F

ATACGGGAGCCAACACCACGCCCCCCCTCACTACTGTCCCGCCCCCCGCCGTGGAGAGCAGGTGTGACGGAT

DE4-7R

ATCCGTCACACCTGCTCTCCACGGCGGGGGGCGGGACAGTAGTGAGGGGGGGCGTGGTGTTGGCTCCCGTAT

DE4-9Fa

ATACGGGAGCCAACACCACCGGCCAACGAAAGACCTCGCTCACTAGACACCCCTAGAGCAGGTGTGACGGAT

DE4-9Fb

ATACGGGAGCCAACACCACCAGAAAATCAATATAACAACGTATGCTGGCTCCGAGAGCAGGTGTGACGGAT

DE4-9Ra

ATCCGTCACACCTGCTCTAGGGGTGTCTAGTGAGCGAGGTCTTTCGTTGGCCGGTGGTGTTGGCTCCCGTAT

DE4-9Rb

ATCCGTCACACCTGCTCTCGGAGCCAGCATACGTTGTTATATTGATTTTCTGGTGGTGTTGGCTCCCGTAT

**Tick-borne Encephalitis Virus (TBEV) Envelope Protein**

TBEV-1F

ATACGGGAGCCAACACCACTCGGCACCGCCCTTCCGTATCGGCGAGTAACGTACAGAGCAGGTGTGACGGAT

TBEV-1R

ATCCGTCACACCTGCTCTGTACGTTACTCGCCGATACGGAAGGGCGGTGCCGAGTGGTGTTGGCTCCCGTAT

TBEV-2F

ATACGGGAGCCAACACCACCGCAGGAGTCCATCAGGGGTTGGCAGTCAGCGCTCAGAGCAGGTGTGACGGAT

TBEV-2R

ATCCGTCACACCTGCTCTGAGCGCTGACTGCCAACCCCTGATGGACTCCTGCGGTGGTGTTGGCTCCCGTAT

TBEV-3F

ATACGGGAGCCAACACCACACGAACGGAGTGCACCGGGGAAGATACTCCAACGCAGAGCAGGTGTGACGGAT

TBEV-3R

ATCCGTCACACCTGCTCTGCGTTGGAGTATCTTCCCCGGTGCACTCCGTTCGTGTGGTGTTGGCTCCCGTAT

TBEV-4F

ATACGGGAGCCAACACCAGCCAATTATACAGGTAGGTCAAAAAAGTTTAGGGGAAGAGCAGGTGTGACGGAT

TBEV-4R

ATCCGTCACACCTGCTCTTCCCCTAAACTTTTTTGACCTACCTGTATAATTGGCTGGTGTTGGCTCCCGTAT

TBEV-5F

ATACGGGAGCCAACACCACCATGCCCCTTTAAGTGAATAGACTAGTGGCCGTTGAGAGCAGGTGTGACGGAT

TBEV-5R

ATCCGTCACACCTGCTCTCAACGGCCACTAGTCTATTCACTTAAAGGGGCATGGTGGTGTTGGCTCCCGTAT

TBEV-6F

ATACGGGAGCCAACACCAGAGACGTCTTAGCCTCGCGATCCCGTCCGTTGGCCCAGAGCAGGTGTGACGGAT

TBEV-6R

ATCCGTCACACCTGCTCTGGGCCAACGGACGGGATCGCGAGGCTAAGACGTCTCTGGTGTTGGCTCCCGTAT

TBEV-7F

ATACGGGAGCCAACACCATCCTCCCGCGACGTCTGGAGAACAGCCTCTACTTTAAGAGCAGGTGTGACGGAT

TBEV-7R

ATCCGTCACACCTGCTCTTAAAGTAGAGGCTGTTCTCCAGACGTCGCGGGAGGATGGTGTTGGCTCCCGTAT

TBEV-8F

ATACGGGAGCCAACACCACACACAGAGCGCCATGGACTCAGTCAGATGTGATGTAGAGCAGGTGTGACGGAT

TBEV-8R

ATCCGTCACACCTGCTCTACATCACATCTGACTGAGTCCATGGCGCTCTGTGTGTGGTGTTGGCTCCCGTAT

TBEV-10F

ATACGGGAGCCAACACCATTGACTTGGCCGTCTCTGACCCCTAGCACCCCTCGCAGAGCAGGTGTGACGGAT

TBEV-10R

ATCCGTCACACCTGCTCTGCGAGGGGTGCTAGGGGTCAGAGACGGCCAAGTCAATGGTGTTGGCTCCCGTAT

**West Nile Virus Envelope (WNV) Protein**

WNV -1F

ATACGGGAGCCAACACCAATAGATGGATAAGGGGGAAACTGCCATTCGGTTAGTAGAGCAGGTGTGACGGAT

WNV -1R

ATCCGTCACACCTGCTCTACTAACCGAATGGCAGTTTCCCCCTTATCCATCTATTGGTGTTGGCTCCCGTAT

WNV - 2F

ATACGGGAGCCAACACCAGATTGAAGCTCAAGCCTAAAGGTGACCAAAGGTAGAAGAGCAGGTGTGACGGAT

WNV – 2R

ATCCGTCACACCTGCTCTTCTACCTTTGGTCACCTTTAGGCTTGAGCTTCAATCTGGTGTTGGCTCCCGTAT

**WNV -3/7/11F**

ATACGGGAGCCAACACCACAGGAGAGGCAGTAAAAGGGTTGGCTGCCTGGGTAGAGAGCAGGTGTGACGGAT

**WNV – 3/7/11R**

ATCCGTCACACCTGCTCTCTACCCAGGCAGCCAACCCTTTTACTGCCTCTCCTGTGGTGTTGGCTCCCGTAT

WNV – 4Fa

ATACGGGAGCCAACACCATTATTACTGAGCTGTGCGCCGCTACCTGCCTAGATTAGAGCAGGTGTGACGGAT

WNV – 4Ra

ATCCGTCACACCTGCTCTAATCTAGGCAGGTAGCGGCGCACAGCTCAGTAATAATGGTGTTGGCTCCCGTAT

WNV - 4Fb

ATACGGGAGCCAACACCAATGCGGGCTTCCTACTCCAACCCAGGACCTTCACCAGAGCAGGTGTGACGGAT

WNV – 4Rb

ATCCGTCACACCTGCTCTGGTGAAGGTCCTGGGTTGGAGTAGGAAGCCCGCATTGGTGTTGGCTCCCGTAT

WNV – 5F

ATACGGGAGCCAACACCACGCGGCTGTCTATGACCGGGCTTGTTGTTTCTGCTAAGAGCAGGTGTGACGGAT

WNV – 5R

ATCCGTCACACCTGCTCTTAGCAGAAACAACAAGCCCGGTCATAGACAGCCGCGTGGTGTTGGCTCCCGTAT

WNV – 8F

ATACGGGAGCCAACACCACGAGGATTACAACTTTATGCGTGCAACCAGACACCAAGAGCAGGTGTGACGGAT

WNV – 8R

ATCCGTCACACCTGCTCTTGGTGTCTGGTTGCACGCATAAAGTTGTAATCCTCGTGGTGTTGGCTCCCGTAT

WNV – 9F

ATACGGGAGCCAACACCACCTACAGATCCGCGAACCAGCCGACTACTCGTCCACAGAGCAGGTGTGACGGAT

WNV - 9R

ATCCGTCACACCTGCTCTGTGGACGAGTAGTCGGCTGGTTCGCGGATCTGTAGGTGGTGTTGGCTCCCGTAT

WNV – 10F (59 bases)

ATACGGGAGCCAACACCACAGCTGATATTGGATGGTCCGGCAGAGCAGGTGTGACGGAT

WNV – 10R (59 bases)

ATCCGTCACACCTGCTCTGCCGGACCATCCAATATCAGCTGTGGTGTTGGCTCCCGTAT

WNV – 12F

ATACGGGAGCCAACACCAAGCTCTCACGTGACACAGTGCTCCGCCGTCAAAATGAGAGCAGGTGTGACGGAT

WNV – 12R

ATCCGTCACACCTGCTCTCATTTTGACGGCGGAGCACTGTGTCACGTGAGAGCTTGGTGTTGGCTCCCGTAT

WNV – 13F

ATACGGGAGCCAACACCATCCCGCGCCCACTGCTTGTCACCTCTTAGCCCCCGCAGAGCAGGTGTGACGGAT

WNV – 13R

ATCCGTCACACCTGCTCTGCGGGGGCTAAGAGGTGACAAGCAGTGGGCGCGGGATGGTGTTGGCTCCCGTAT

WNV – 14F

ATACGGGAGCCAACACCAACCCAATAAACTTATTGGACCTACGCTTTGATGATTAGAGCAGGTGTGACGGAT

WNV – 14R

ATCCGTCACACCTGCTCTAATCATCAAAGCGTAGGTCCAATAAGTTTATTGGGTTGGTGTTGGCTCCCGTAT

WNV – 15F

ATACGGGAGCCAACACCACACTGCATCCCTCTACCGTACTTACATTCCTGACATAGAGCAGGTGTGACGGAT

WNV – 15R

ATCCGTCACACCTGCTCTATGTCAGGAATGTAAGTACGGTAGAGGGATGCAGTGTGGTGTTGGCTCCCGTAT

WNV – 16F

ATACGGGAGCCAACACCATGTCAGGACCTCCATCGCCCGGGCCCGCCGCCGCTGAGAGCAGGTGTGACGGAT

WNV – 16R

ATCCGTCACACCTGCTCTCAGCGGCGGCGGGCCCGGGCGATGGAGGTCCTGACATGGTGTTGGCTCCCGTAT

WNV – 18F

ATACGGGAGCCAACACCACCCCGTCGCCAAGCACTTGGCTGGGCTCTAACGGCCAGAGCAGGTGTGACGGAT

WNV – 18R

ATCCGTCACACCTGCTCTGGCCGTTAGAGCCCAGCCAAGTGCTTGGCGACGGGGTGGTGTTGGCTCCCGTAT

WNV – 19F (59 bases)

ATACGGGAGCCAACACCACAGCTGATATCGGATGGTCCGGCAGAGCAGGTGTGACGGAT

WNV – 19R (59 bases)

ATCCGTCACACCTGCTCTGCCGGACCATCCGATATCAGCTGTGGTGTTGGCTCCCGTAT

WNV – 20F

ATACGGGAGCCAACACCACCCGTGGCCTTCACCCAGCCAGGGGCCCCGTCTCTGAGAGCAGGTGTGACGGAT

WNV – 20R

ATCCGTCACACCTGCTCTCAGAGACGGGGCCCCTGGCTGGGTGAAGGCCACGGGTGGTGTTGGCTCCCGTAT
