# Supplementary material for: Development, screening, and analysis of DNA aptamer libraries potentially useful for diagnosis and passive immunity of arboviruses
Source: BMC Res Notes. 2012 Nov 13;5:633. doi: 10.1186/1756-0500-5-633 (PMC3517355; doi:10.1186/1756-0500-5-633)
Supplement: Additional file 2 — Figure S1. Secondary structures of the top ten ELASA ranked Chikungunya aptamers. Figure S2. Secondary structures of the top ten ELASA ranked Crimean-Congo Hemorrhagic Fever (CCHF) Altamura Gn611 aptamers. Figure S3. Secondary structures of the top ten ELASA ranked CCHF 11E7a aptamers. Figure S4. Secondary structures of the top ten ELASA ranked CCHF 11E7b aptamers. Figure S5. Secondary structures of the top ten ELASA ranked CCHF 11E7c aptamers. Figure S6. Secondary structures of the top ten ELASA ranked CCHF Drosdov strain whole virus-developed aptamers. Figure S7. Secondary structures of consensus CCHF IbAr 10200 strain whole virus-developed aptamers in the entire aptamer library. Note that the reverse sequence contains the most common ACGGGTCCGGACA sequence segment (underlined) in its structure as well. Figure S8. Secondary structures of the top ten ELASA ranked dengue serotype 1 aptamers. Figure S9. Secondary structures of the top ten ELASA ranked dengue serotype 2 aptamers. Figure S10. Secondary structures of the top ten ELASA ranked dengue serotype 3 aptamers. Figure S11. Secondary structures of the top ten ELASA ranked dengue serotype 4 aptamers. Figure S12. Secondary structures of the top ten ELASA ranked Tick-borne Encephalitis Virus (TBEV) aptamers. Figure S13. Secondary structures of the top ten ELASA ranked West Nile Virus (WNV) aptamers. [file 1756-0500-5-633-S2.ppt]

## Slide 1
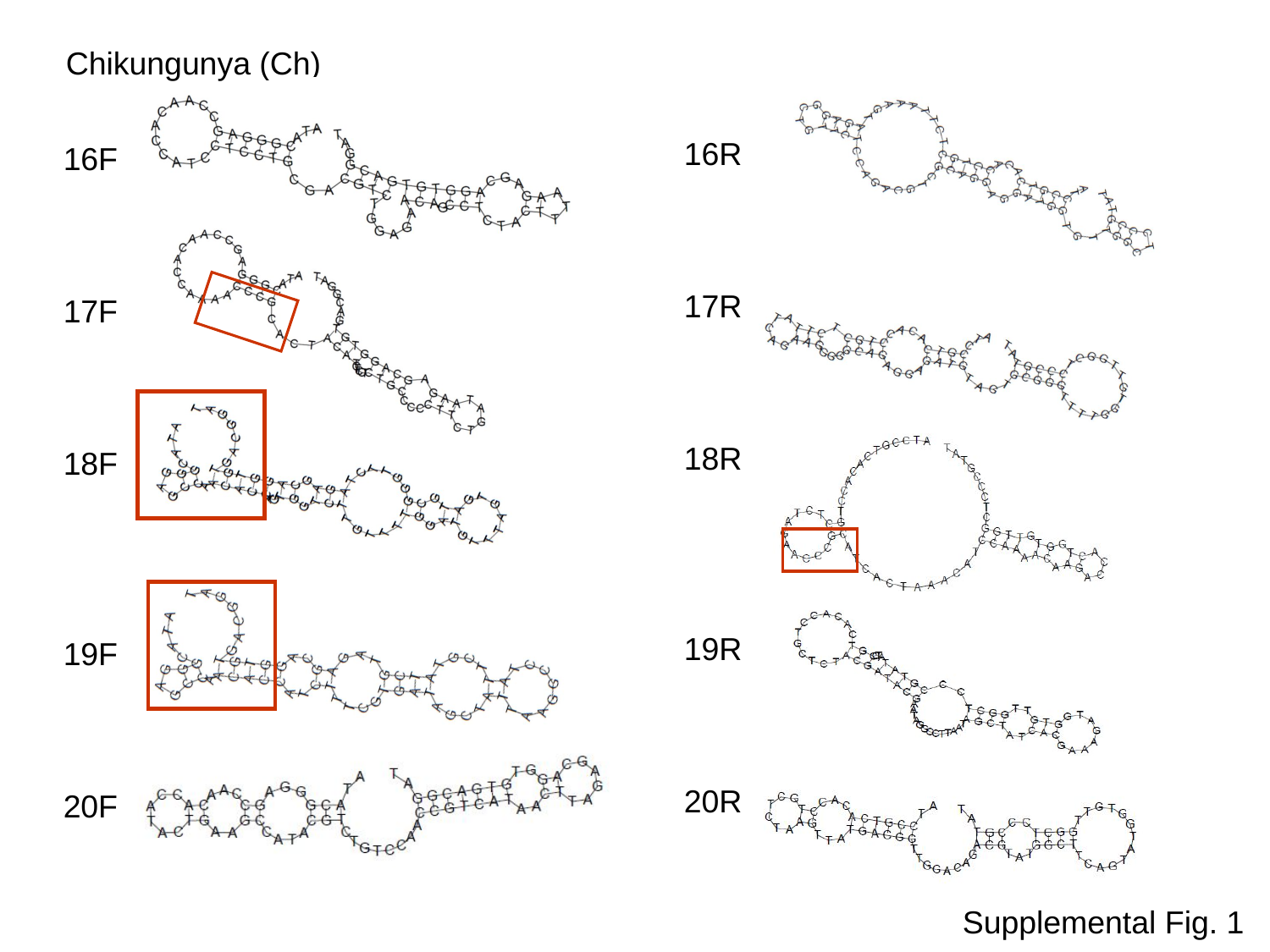

Chikungunya (Ch)
16R
17R
18R
19R
20R
16F
17F
18F
19F
20F
Supplemental Fig. 1

## Slide 2
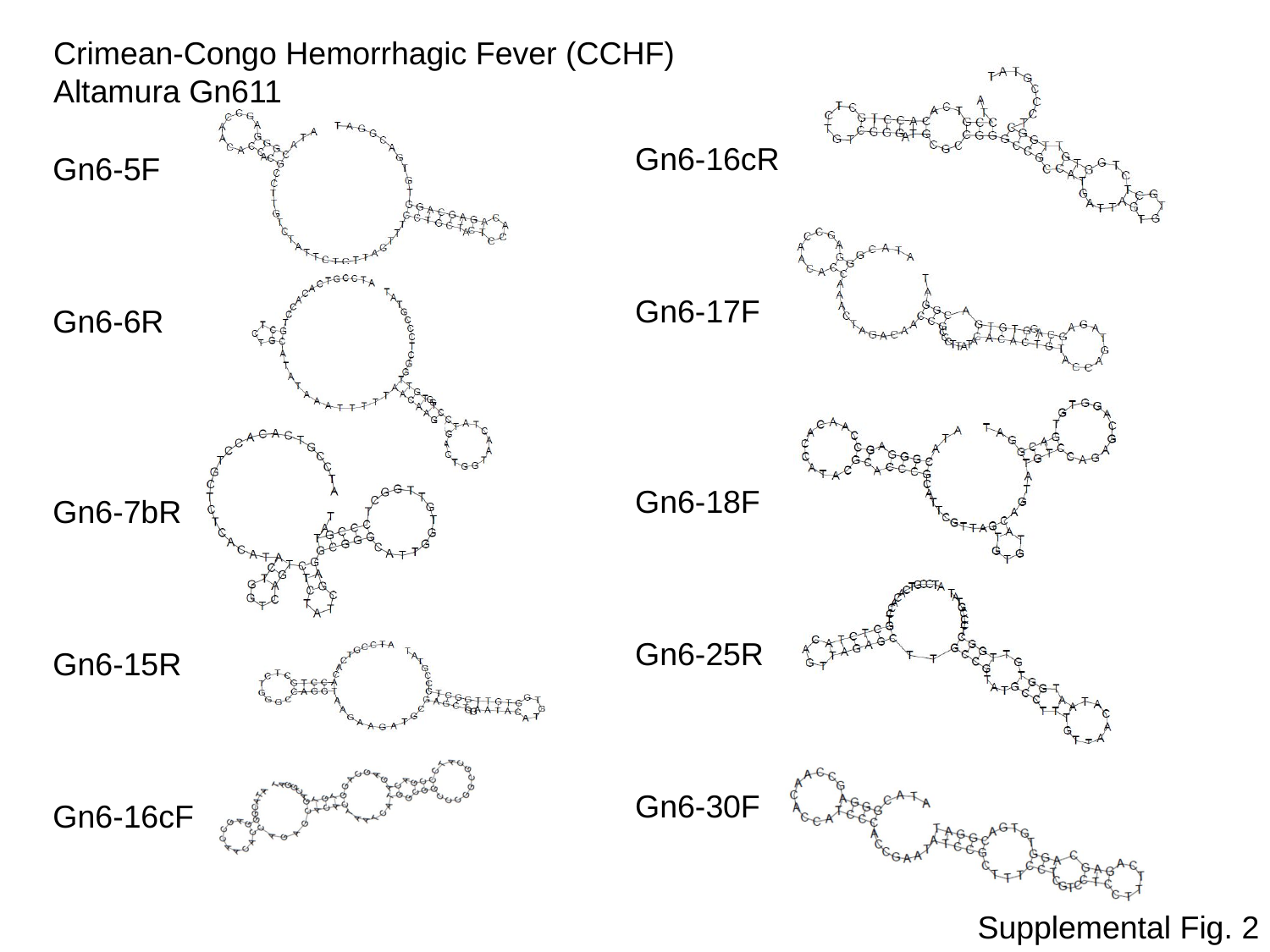

Crimean-Congo Hemorrhagic Fever (CCHF)
Altamura Gn611
Gn6-16cR
Gn6-17F
Gn6-18F
Gn6-25R
Gn6-30F
Gn6-5F
Gn6-6R
Gn6-7bR
Gn6-15R
Gn6-16cF
Supplemental Fig. 2

## Slide 3
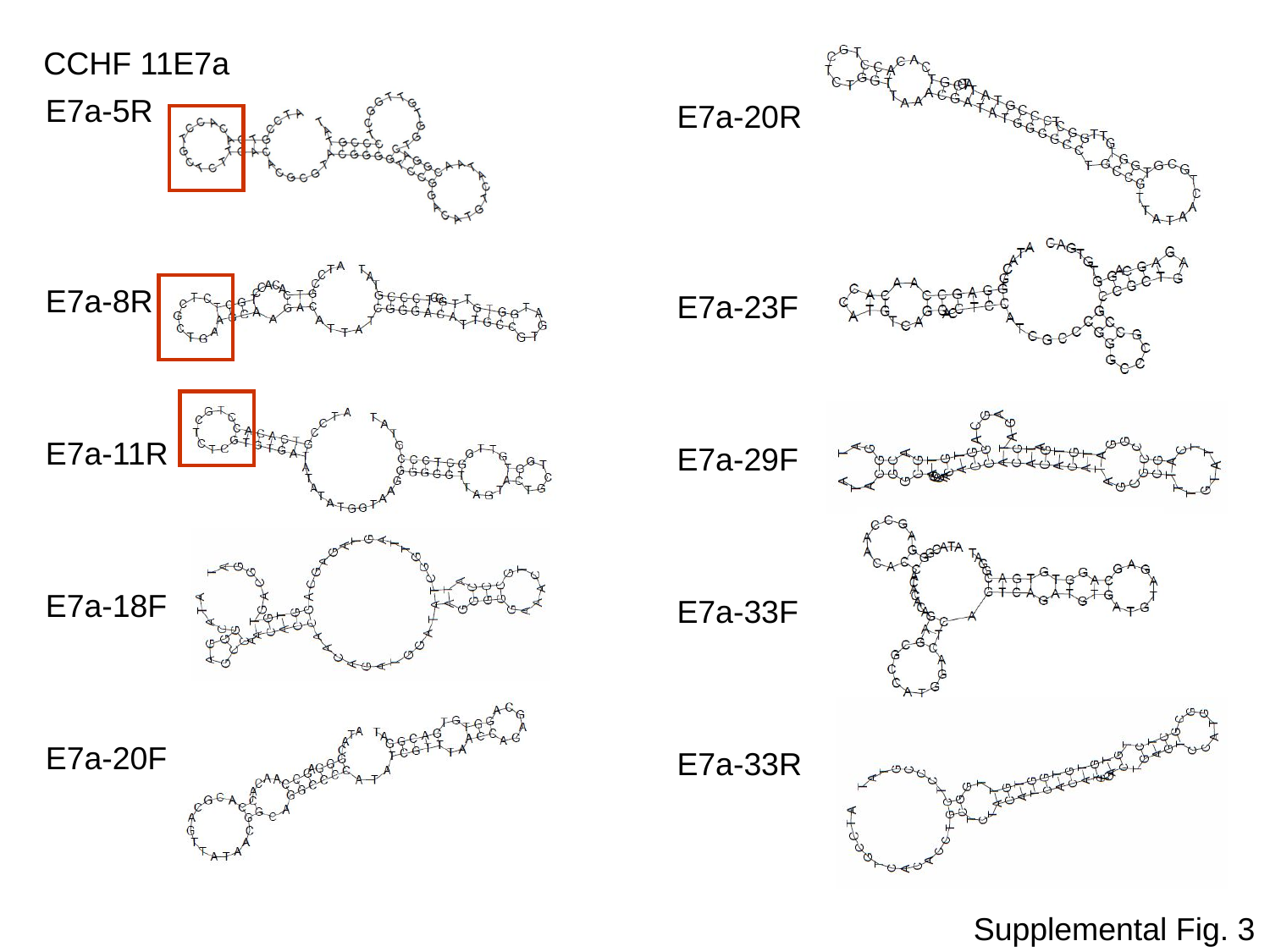

CCHF 11E7a
E7a-5R
E7a-8R
E7a-11R
E7a-18F
E7a-20F
E7a-20R
E7a-23F
E7a-29F
E7a-33F
E7a-33R
Supplemental Fig. 3

## Slide 4
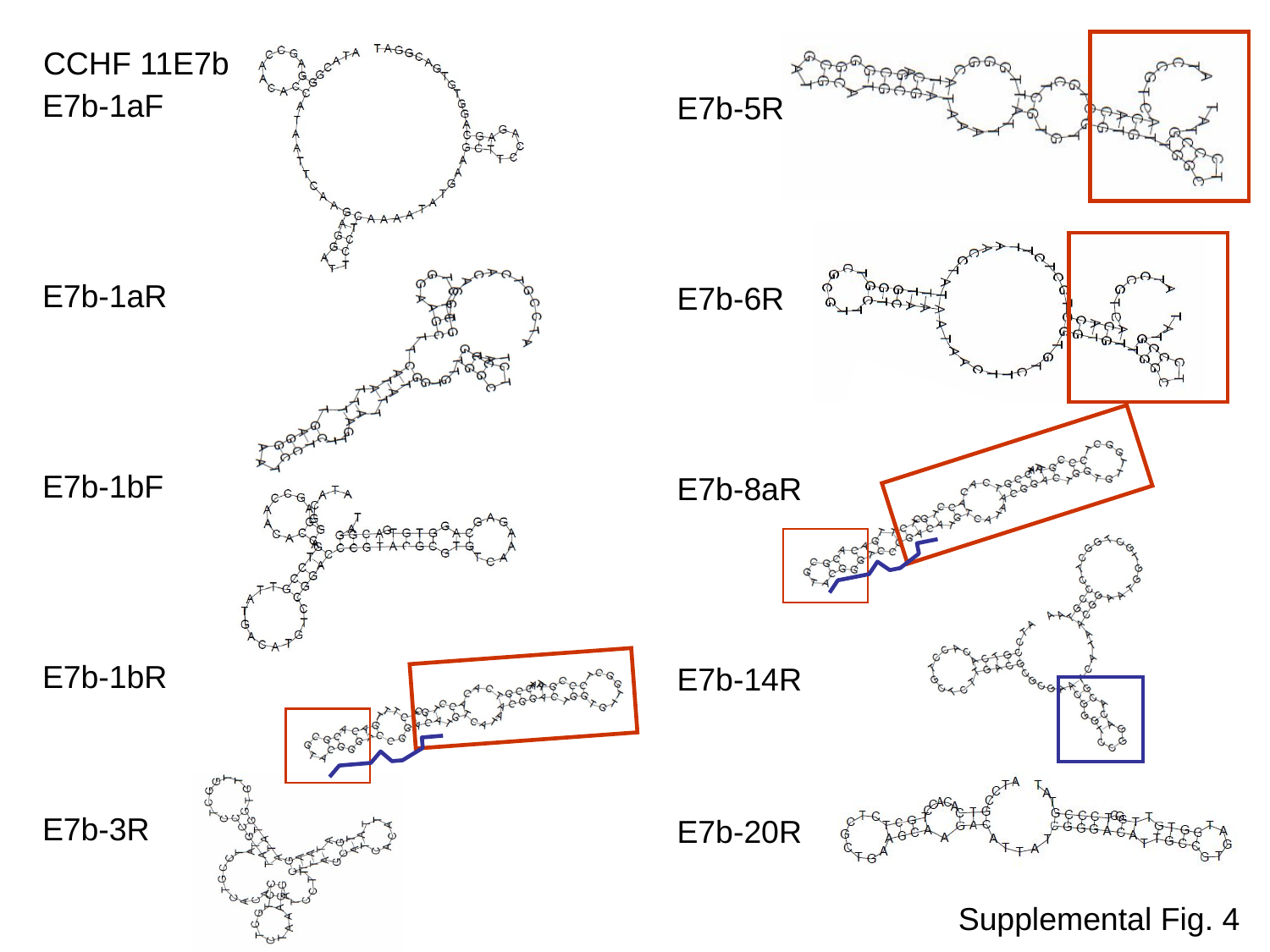

CCHF 11E7b
E7b-1aF
E7b-1aR
E7b-1bF
E7b-1bR
E7b-3R
E7b-5R
E7b-6R
E7b-8aR
E7b-14R
E7b-20R
Supplemental Fig. 4

## Slide 5
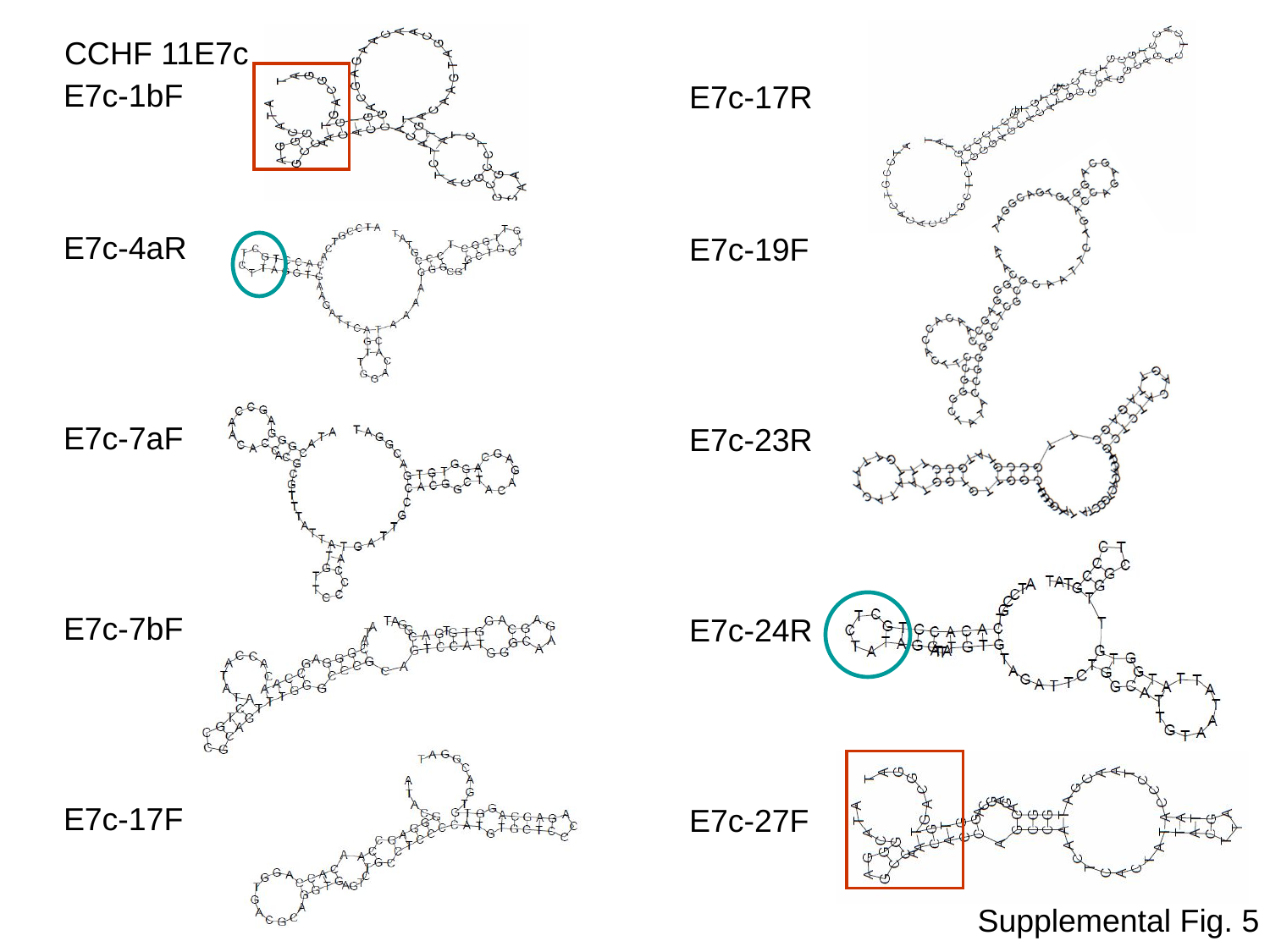

CCHF 11E7c
E7c-1bF
E7c-4aR
E7c-7aF
E7c-7bF
E7c-17F
E7c-17R
E7c-19F
E7c-23R
E7c-24R
E7c-27F
Supplemental Fig. 5

## Slide 6
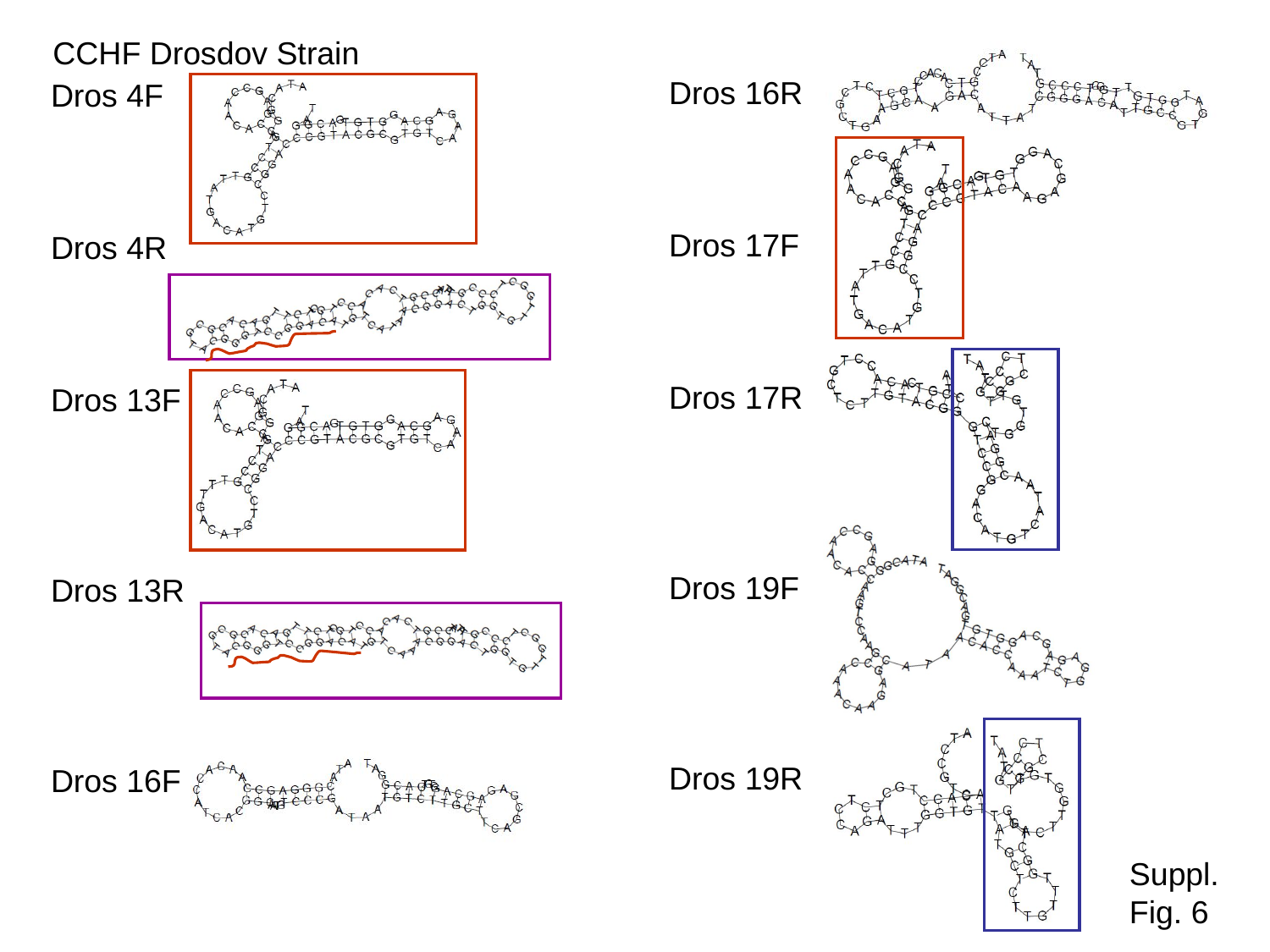

CCHF Drosdov Strain
Dros 16R
Dros 17F
Dros 17R
Dros 19F
Dros 19R
Dros 4F
Dros 4R
Dros 13F
Dros 13R
Dros 16F
Suppl.
Fig. 6

## Slide 7
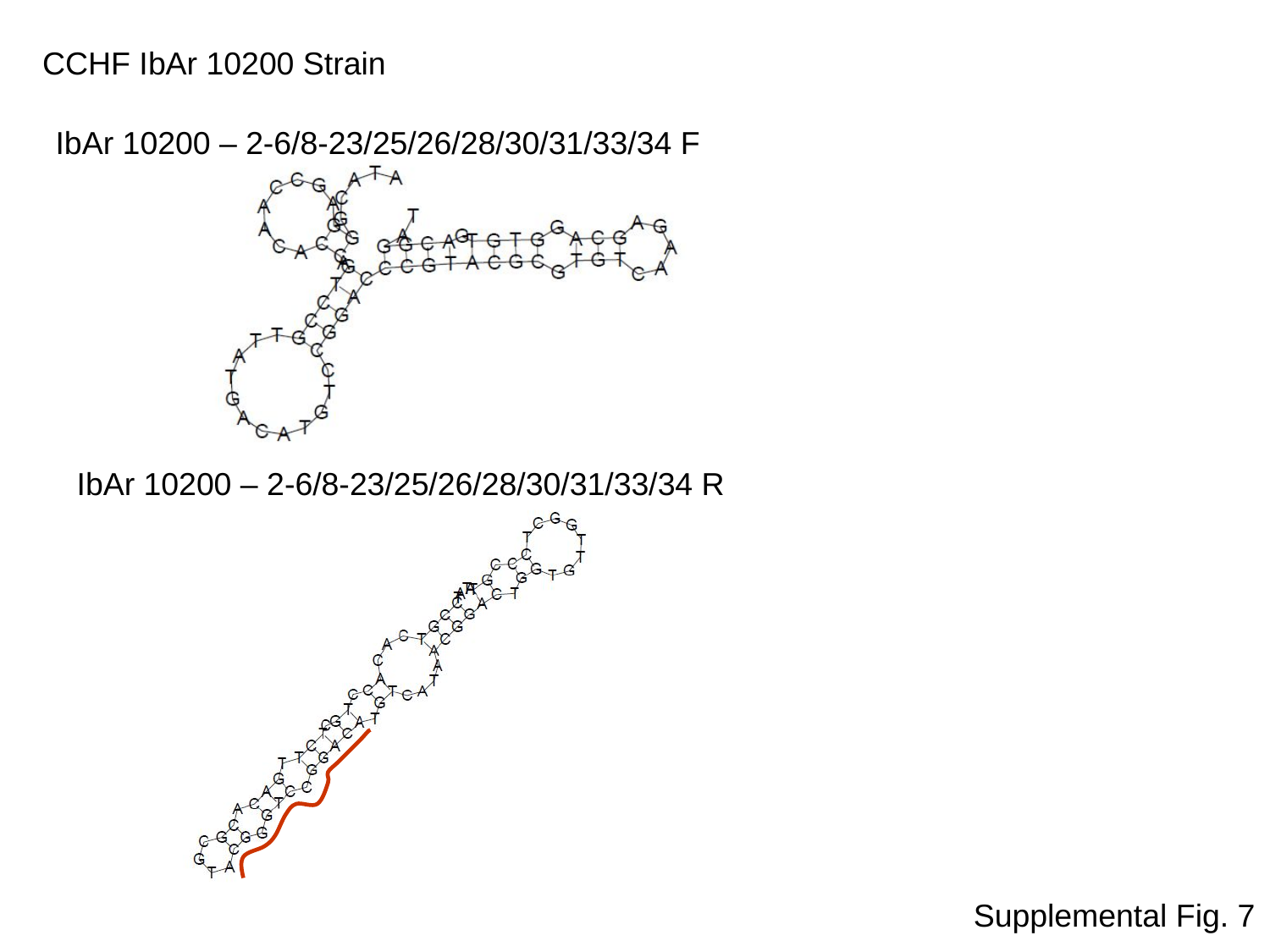

CCHF IbAr 10200 Strain
IbAr 10200 – 2-6/8-23/25/26/28/30/31/33/34 F
IbAr 10200 – 2-6/8-23/25/26/28/30/31/33/34 R
Supplemental Fig. 7

## Slide 8
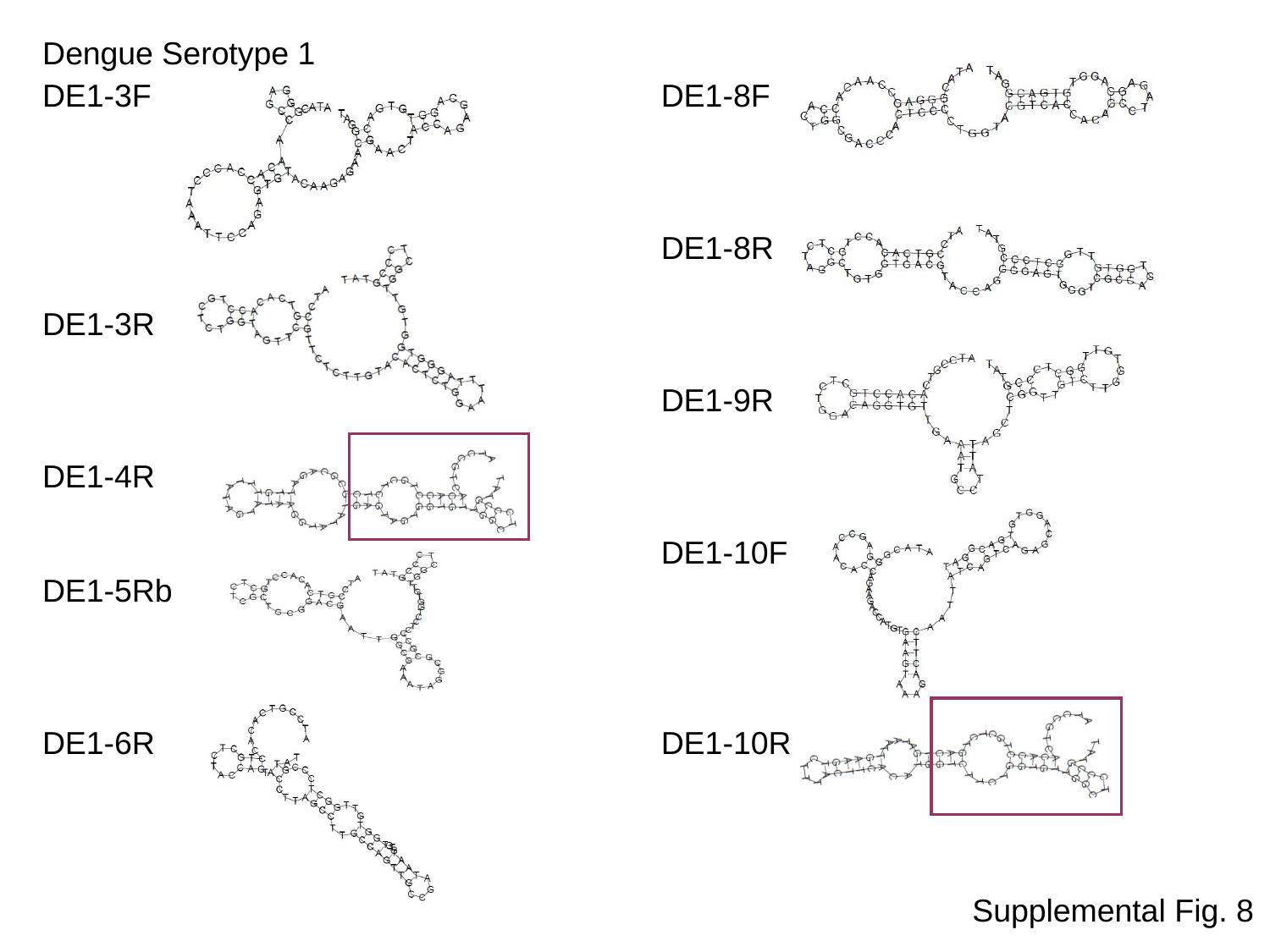

Dengue Serotype 1
DE1-3F
DE1-3R
DE1-4R
DE1-5Rb
DE1-6R
DE1-8F
DE1-8R
DE1-9R
DE1-10F
DE1-10R
Supplemental Fig. 8

## Slide 9
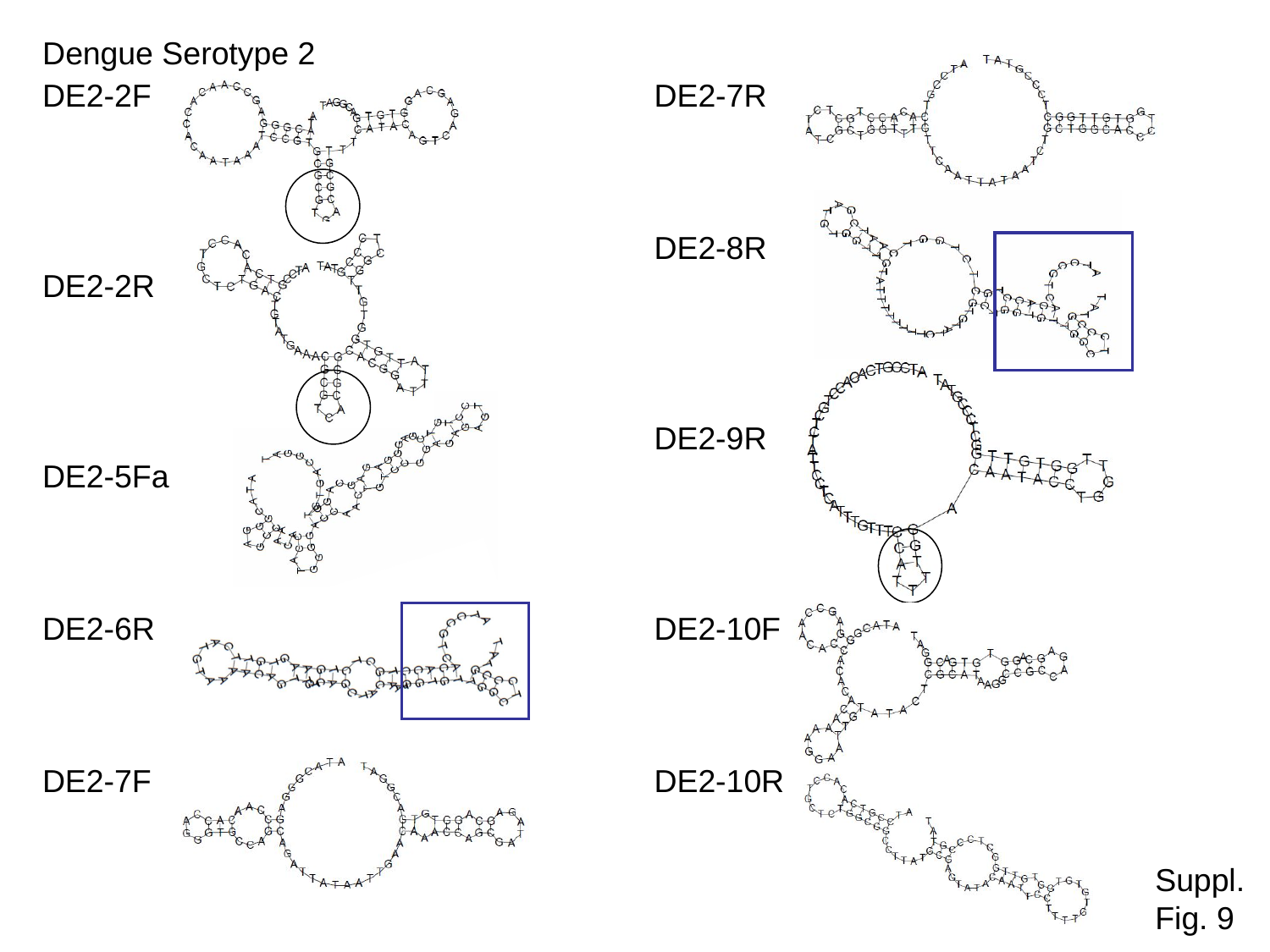

Dengue Serotype 2
DE2-2F
DE2-2R
DE2-5Fa
DE2-6R
DE2-7F
DE2-7R
DE2-8R
DE2-9R
DE2-10F
DE2-10R
Suppl.
Fig. 9

## Slide 10
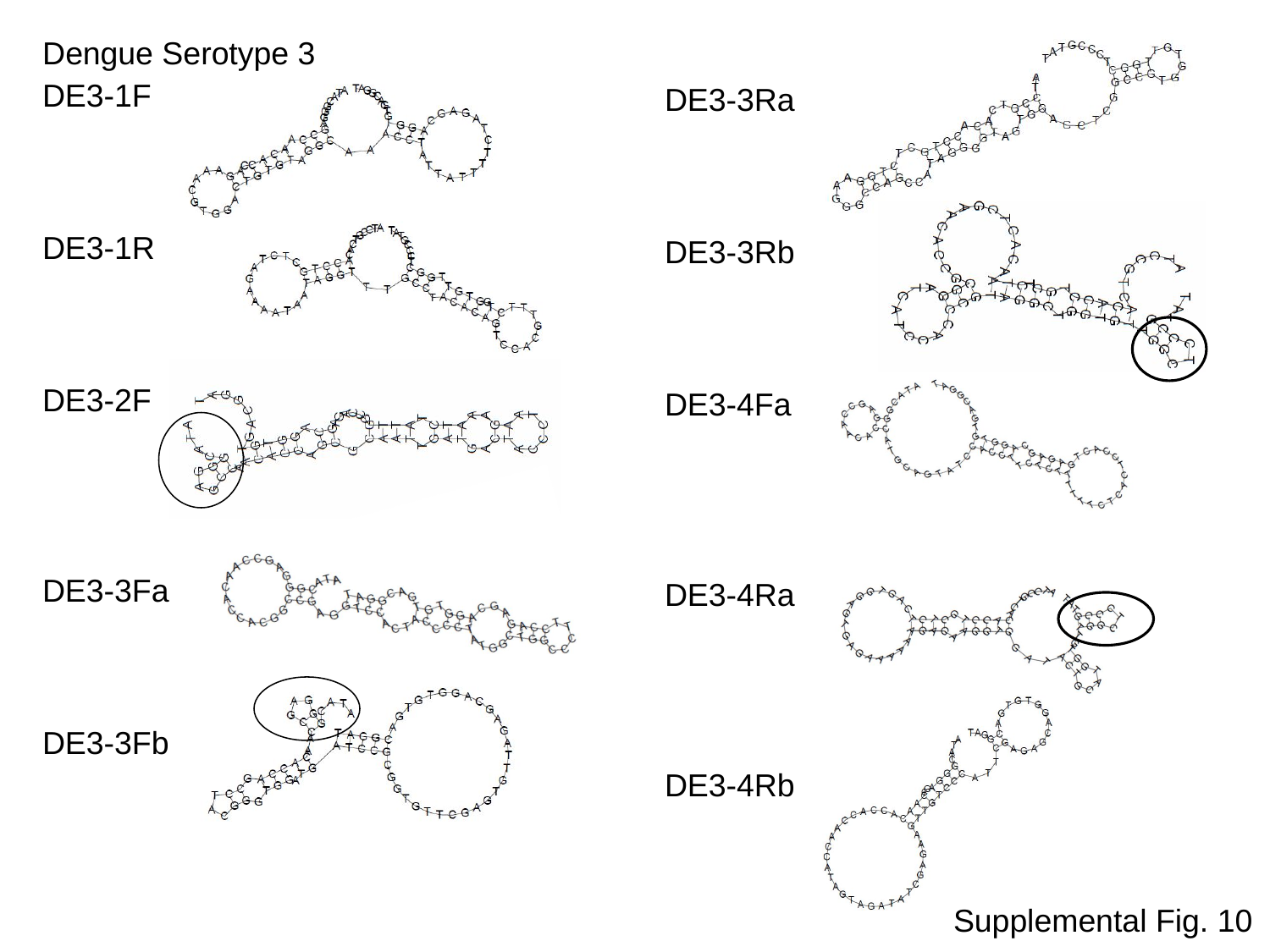

Dengue Serotype 3
DE3-1F
DE3-1R
DE3-2F
DE3-3Fa
DE3-3Fb
DE3-3Ra
DE3-3Rb
DE3-4Fa
DE3-4Ra
DE3-4Rb
Supplemental Fig. 10

## Slide 11
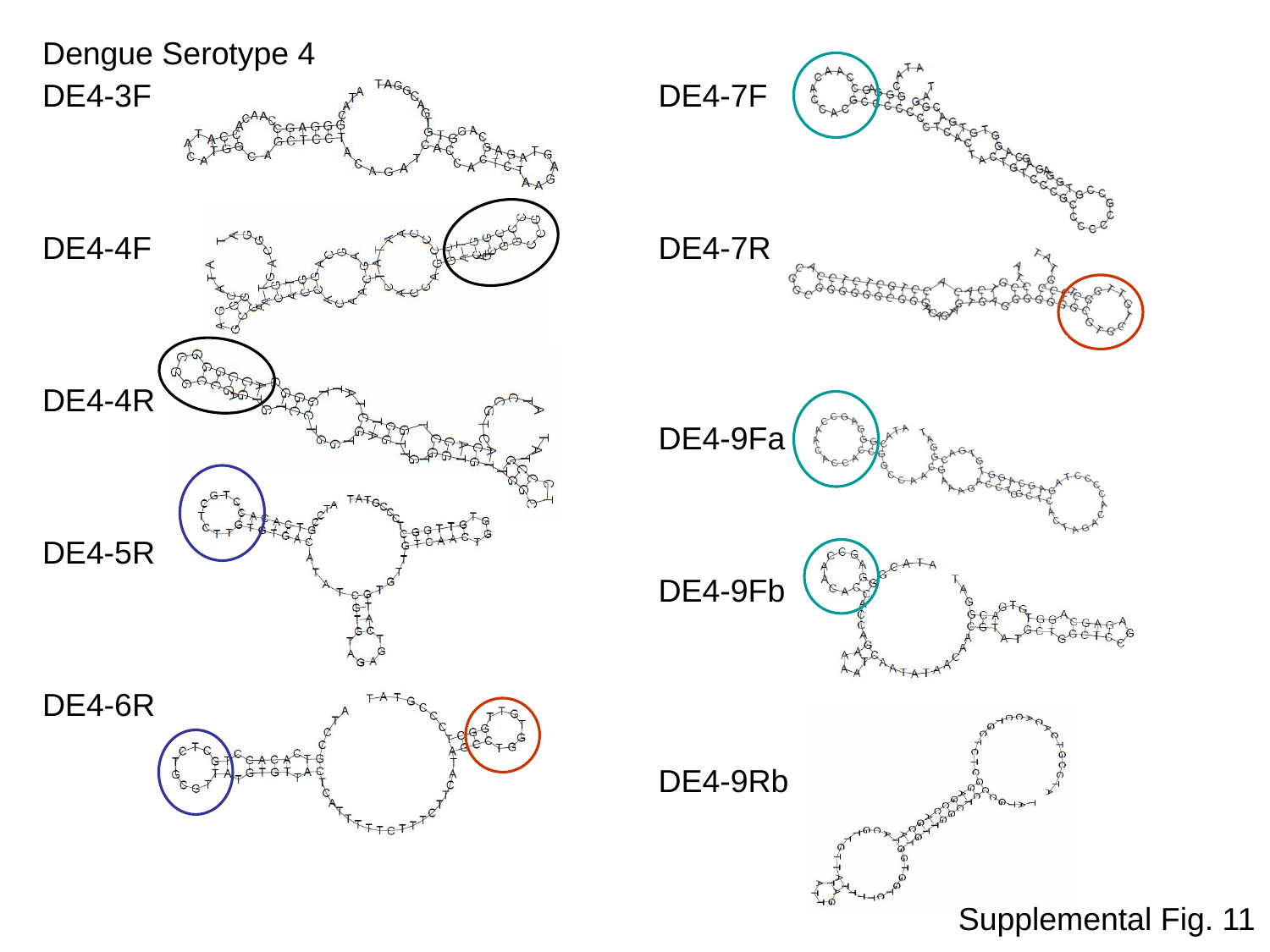

Dengue Serotype 4
DE4-3F
DE4-4F
DE4-4R
DE4-5R
DE4-6R
DE4-7F
DE4-7R
DE4-9Fa
DE4-9Fb
DE4-9Rb
Supplemental Fig. 11

## Slide 12
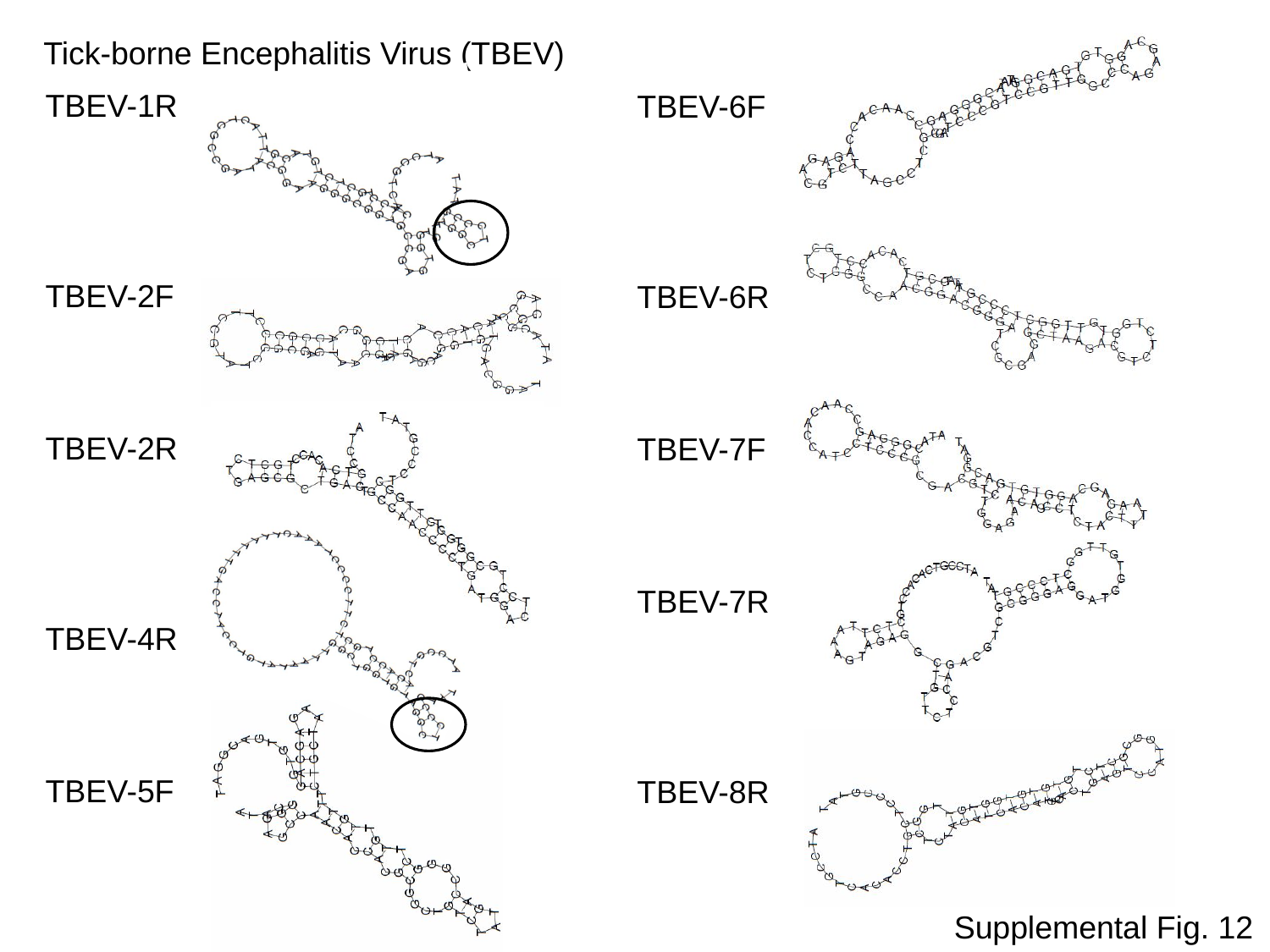

Tick-borne Encephalitis Virus (TBEV)
TBEV-1R
TBEV-2F
TBEV-2R
TBEV-4R
TBEV-5F
TBEV-6F
TBEV-6R
TBEV-7F
TBEV-7R
TBEV-8R
Supplemental Fig. 12

## Slide 13
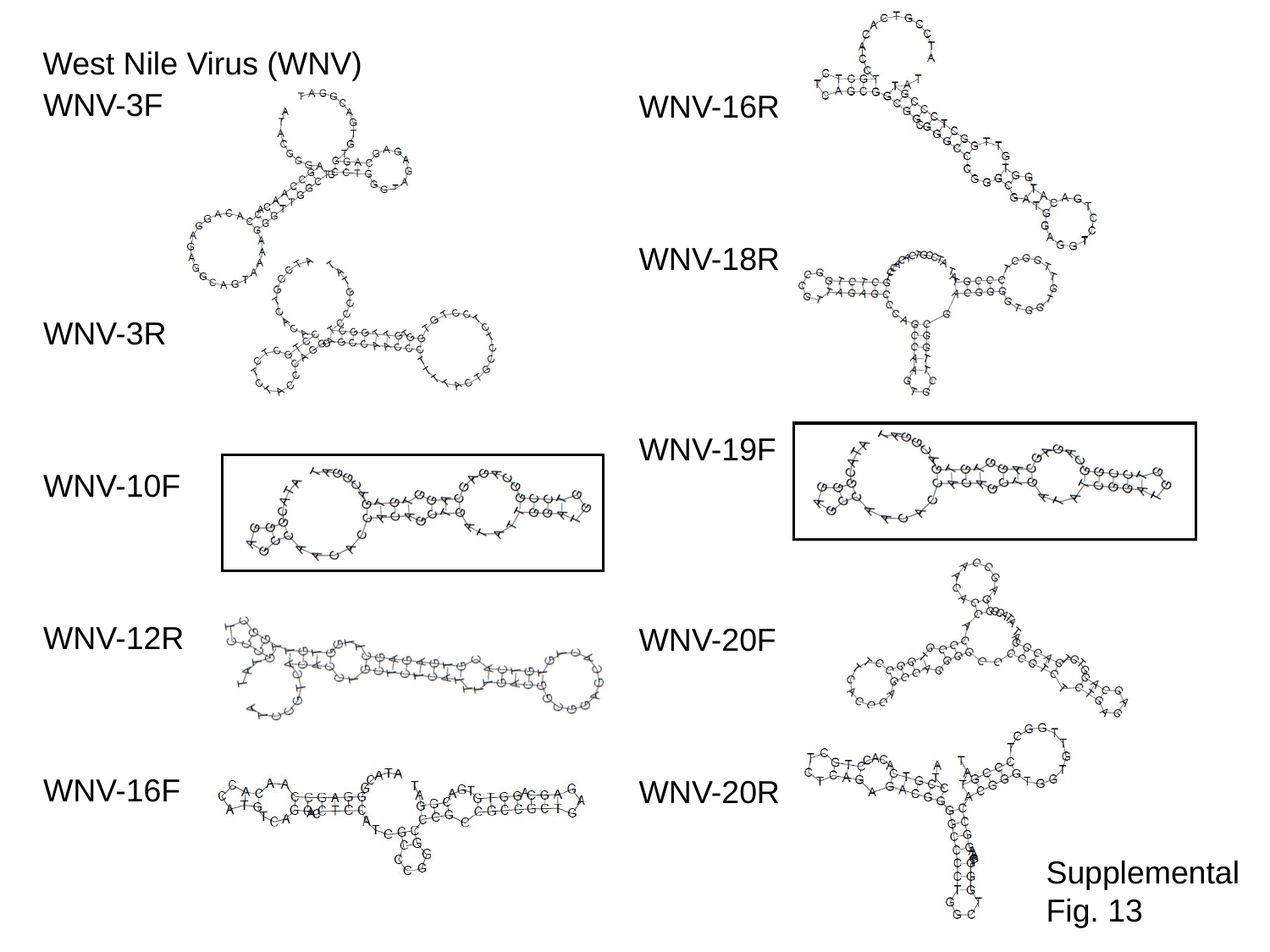

West Nile Virus (WNV)
WNV-3F
WNV-3R
WNV-10F
WNV-12R
WNV-16F
WNV-16R
WNV-18R
WNV-19F
WNV-20F
WNV-20R
Supplemental
Fig. 13
